# Effects of Timber Harvests and Silvicultural Edges on Terrestrial Salamanders

**DOI:** 10.1371/journal.pone.0114683

**Published:** 2014-12-17

**Authors:** Jami E. MacNeil, Rod N. Williams

**Affiliations:** Department of Forestry and Natural Resources, Purdue University, West Lafayette, Indiana, United States of America; Clemson University, United States of America

## Abstract

Balancing timber production and conservation in forest management requires an understanding of how timber harvests affect wildlife species. Terrestrial salamanders are useful indicators of mature forest ecosystem health due to their importance to ecosystem processes and sensitivity to environmental change. However, the effects of timber harvests on salamanders, though often researched, are still not well understood. To further this understanding, we used artificial cover objects to monitor the relative abundance of terrestrial salamanders for two seasons (fall and spring) pre-harvest and five seasons post-harvest in six forest management treatments, and for three seasons post-harvest across the edge gradients of six recent clearcuts. In total, we recorded 19,048 encounters representing nine species of salamanders. We observed declines in mean encounters of eastern red-backed salamanders (*Plethodon cinereus*) and northern slimy salamanders (*P. glutinosus*) from pre- to post-harvest in group selection cuts and in clearcuts. However, we found no evidence of salamander declines at shelterwoods and forested sites adjacent to harvests. Edge effects induced by recent clearcuts influenced salamanders for approximately 20 m into the forest, but edge influence varied by slope orientation. Temperature, soil moisture, and canopy cover were all correlated with salamander counts. Our results suggest silvicultural techniques that remove the forest canopy negatively affect salamander relative abundance on the local scale during the years immediately following harvest, and that the depth of edge influence of clearcuts on terrestrial salamanders is relatively shallow (<20 m). Small harvests (<4 ha) and techniques that leave the forest canopy intact may be compatible with maintaining terrestrial salamander populations across a forested landscape. Our results demonstrate the importance of examining species-specific responses and monitoring salamanders across multiple seasons and years. Long-term monitoring will be necessary to understand the full impacts of forest management on terrestrial salamanders.

## Introduction

Forest management in the US has traditionally focused on the production of timber, but recent decades have seen a shift toward ecosystem-based management, an approach that seeks to balance timber production with the maintenance of biodiversity and ecological function [1]–[3]. This approach requires knowledge of how forest systems respond to different management techniques [2], [4]. All forms of logging alter forest structure, resulting in changes to physical factors (e.g., temperature, humidity, wind exposure, light intensity; [5], [6]) that affect microhabitat and resource availability both within the harvest opening and across some distance into the adjacent forest [7], [8]. While these changes are beneficial for some species [9], [10], they can pose challenges to forest-dependent species, with consequences for resource availability, cover, thermoregulation, movement, and exposure to predators (e.g., [11]–[14]). Understanding the effects of different timber harvest techniques on sensitive species is an important step in creating ecosystem-based management plans for forest systems.

Terrestrial salamanders of the family *Plethodontidae* are small-bodied, lungless amphibians that typically reside in the leaf litter and soil of the forest floor. They form an integral component of forest nutrient cycles, act as regulators of soil invertebrates, and influence rates of decomposition in the litter layer [15], [16]. Many species are widespread, relatively abundant, and occur in high densities in the eastern United States, with estimates surpassing two per square meter in some forested regions [16]–[19]. Their physiology, small home range and limited dispersal capability make terrestrial salamanders sensitive to changes in physical factors such as temperature, humidity, and soil moisture that are commonly altered by forest management [2], [16], [20]. For these reasons, terrestrial salamanders are considered indicators of mature forest ecosystem health [16] and are an ideal taxon to monitor before and after habitat disturbance.

Previous studies on the effects of forest management on salamander abundance vary widely in sampling technique, duration, spatial extent, and species examined (S1 Table; [21]), and as such, often produce conflicting results and interpretations. Many studies find that clearcuts negatively impact salamander abundance at the local scale [22]–[24] while Renken et al. [25] found no such effect at the landscape scale. Within studies that find negative effects of clearcuts on salamander abundance, estimates of time to population recovery range widely from 15 years to 60+ years (S1 Table; [26]–[28]). The effects of alternative harvest techniques such as shelterwood and group selection cuts have been studied less often than those of clearcuts. Some studies have found group and single-tree selection management reduce salamander counts relative to control sites, though to a lesser extent than clearcuts [29], [30], while still other studies have found no effect of group selection cutting [25], forest thinning [24], or firewood cutting [31] on salamander abundance and species presence. Many studies lack pre-harvest data and suffer from a low number of replicates as well as high spatial and temporal variation in site conditions [21]. Thus, despite the extensive treatment of this topic in the literature, the effects of timber harvests on salamanders, particularly recovery times and the relative impact of different harvest strategies, are still not well understood.

Similarly, the effects of silvicultural edges on terrestrial salamanders are not well known. Microenvironmental variables such as light, temperature, humidity, and vegetative cover may be influenced by a harvest opening for 30 m to >240 m into the adjacent forest, depending on site characteristics and local weather [5], [7], [8]. Few studies have examined edge effects on terrestrial salamanders, and their results are not consistent. In Maine [32] and New Hampshire [33], terrestrial salamander abundance was reduced for 20–35 m into mature forest abutting regenerating clearcuts. In contrast, no relationship was found between the abundance of Plethodontid salamanders and distance to silvicultural edges in Oregon [34] or in northern California [35]. A better understanding of edge effects on sensitive species is critical in planning the optimal size and shape of harvest openings for ecosystem-based management.

We sought to further the understanding of how timber harvests and harvest edges affect different species of terrestrial salamanders. We conducted our study in two parts, first examining the effects of multiple harvest techniques and second investigating the effects of clearcut edges on terrestrial salamanders. To study harvest effects we monitored salamander use of artificial cover objects (ACOs) within six types of forest treatment before and after harvests. To study edge effects we monitored salamander use of ACOs across the edge gradient of six replicated clearcuts. We analyzed the effects of treatments and habitat characteristics in terms of salamander relative abundance and also in terms of abundance and probability of detection. Our results provide insight into the short term (<5 yr) response of terrestrial salamanders to timber harvests and harvest edges. Continued monitoring at these sites will provide much needed understanding of the long-term effects of forest management on these ecologically valuable species.

## Materials and Methods

### Ethics Statement

Data collection on salamanders was carried out in accordance with Purdue Animal Care and Use Committee Protocol #95-019-10 and Indiana Department of Natural Resources Scientific Purpose Licenses and amendments 07-0068 and 10-0076. None of the species encountered in this study are listed as threatened or endangered by the state of Indiana Department of Natural Resources or by the United States Fish and Wildlife Service (USFWS).

### Study area

We conducted research within Morgan-Monroe and Yellowwood State Forests in south-central Indiana. Together, these state forests encompass approximately 19,100 ha. The forest type is a mixture of oak-hickory and beech-maple [36]. The topography is characterized by steep ridges and valleys. Both state forests are managed for hunting, recreation, research, and timber production.

We collected data in nine study areas, hereafter referred to as units, established as part of the Hardwood Ecosystem Experiment (HEE) [37], a large-scale and long-term collaborative study on the ecological effects of timber harvests in Indiana. Each unit consists of a core area ranging from 78–110 ha, surrounded by a buffer area ranging from 219–392 ha. In 2006, units were randomly assigned one of three management types, control, uneven-aged, or even-aged, resulting in three replicates of each type. Control units underwent no cutting during the study. Uneven-aged management units each received eight group selection openings ranging in size from 0.2–2.6 ha (mean 1.1 ha; these are also referred to as patch cuts). The remaining area of uneven-aged management units received single-tree selection harvesting with a target basal area of 16.1–23.0 m^2^/ha. Even-aged management units each received two 4-ha clearcuts and two 4-ha shelterwood harvests. Clearcuts involved removal of all woody stems with diameter at breast height (dbh) >30.48 cm, followed by timber stand improvement that coppiced, felled, or girdled remaining trees >2.54 cm dbh. The shelterwoods are a three-stage system; this study encompasses the preparatory cut, which removed midstory and understory layers (target basal area ≥13.8 m^2^/ha). Harvests were conducted between July 2008 and February 2009.

### Study design

#### Harvest effects

To investigate the effects of harvests, we established 66 artificial cover object (ACO; [38]) grids throughout the nine study units in May of 2007. Cover objects were 30×30×5 cm untreated pine boards. Grids consisted of 30 boards arranged 6×5 with 5-m spacing [39], [40]. We placed boards in direct contact with the soil. We initially placed two grids randomly within each control unit, and added an additional six grids to each control unit in the fall of 2009. Within each uneven-aged management unit, we placed one grid inside each of the eight areas designated for a group opening (see Figure 13 in [37]). Within each even-aged management unit, we placed two grids inside and one grid outside (at least 40 m from the edge; [32]) each of the four areas designated for harvest (see Figure 15 in [37]). One grid meant to be within a shelterwood treatment fell outside actual harvest boundaries and was reclassified as a grid outside a shelterwood. Another grid meant to be within a group selection cut also fell outside actual harvest boundaries and was dropped from analysis. In both cases, a new grid was established within the actual harvest area in fall 2010 (data from both new grids were included in analysis). The ACO grids thus represented six treatments types, here referred to as control (n = 24), group (n = 24), clearcut (n = 12), clearcut adjacent (n = 6), shelterwood (n = 12), and shelterwood adjacent (n = 7).

Compass orientation of a site can influence microhabitat variables [8] and salamander abundance [41]. We attempted to place an equal number of grids on northeast- and southwest-facing slopes, although natural topography sometimes prevented ideal grid placement. For the analysis of variance we broadly categorized grids as northeast-facing (azimuth 321–134, n = 42) or southwest-facing (azimuth 140–315, n = 43). We used these broad azimuth ranges because narrowing them would have either excluded many grids or created widely uneven sample sizes within slope by treatment combinations, resulting in non-estimable least-square means for some post-hoc comparisons (see Statistical Analyses section; [42]). For our N-mixture model analysis however, we were able to categorize grids more narrowly as northeast-facing (azimuth 0–90, n = 27), southeast-facing (azimuth 95–170, n = 11), southwest-facing (azimuth 180–270, n = 28), or northwest-facing (azimuth 282–350, n = 19).

We sampled harvest effect grids during the day (0800 h–1800 h) every two weeks each fall (September–November) and spring (March–May) from September 2007 to May 2011. Grids were sampled three to six times during each sample period (fall or spring in a given year). On each sampling occasion (a single check of a single grid), trained observers lifted all boards in a grid and recorded the number and species of each salamander encountered. Animals were handled only if necessary for species identification and released immediately at the site of capture.

#### Edge effects

**Figure 1:**
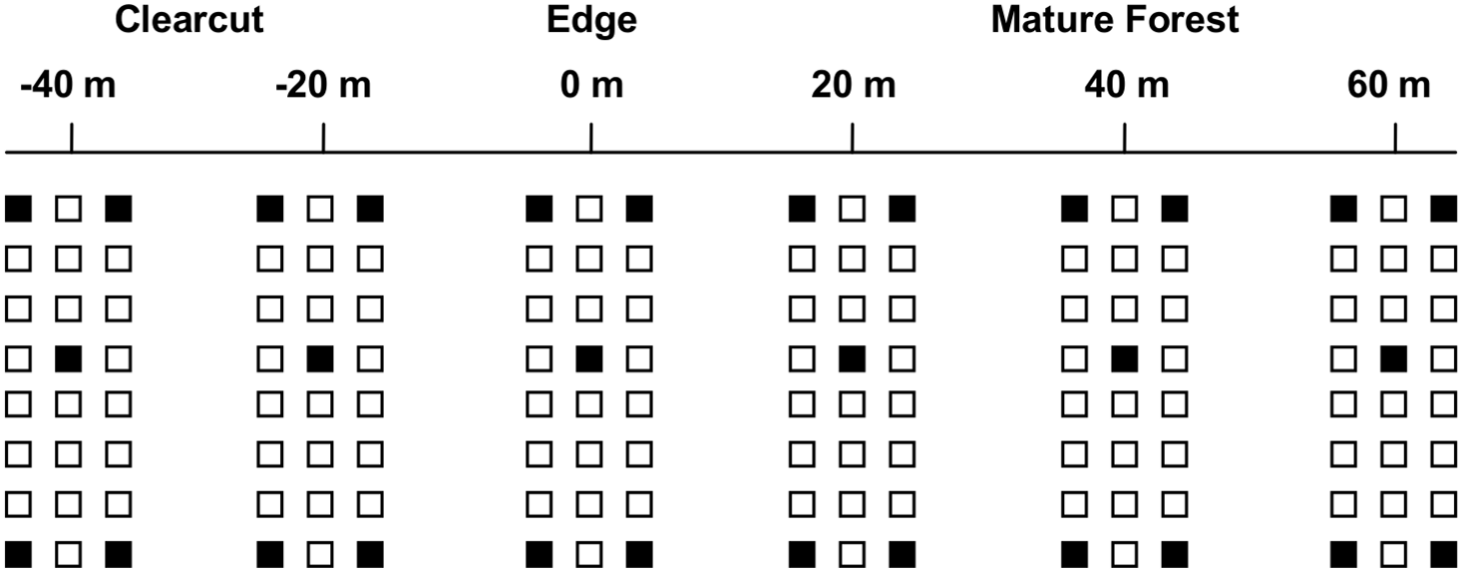
Diagram of edge transect. Edge transects contained six grids of artificial cover objects (ACOs) laid out at 20-m intervals with 3-m spacing between objects. ACOs were solid wood boards, 30×30×5 cm, represented here by open and shaded boxes. Shaded boxes also represent the location of canopy, leaf litter, and soil sampling within the grid.

To study effects of clearcut edges on terrestrial salamanders, we installed ACOs in January 2010 at each of the six clearcuts in the HEE. We placed three transects on northeast-facing slopes and three transects on southwest-facing slopes, in accordance with the general orientation of the clearcut. We placed transects approximately mid-slope and ran them parallel to the contours of the slope, running 40 m into the clearcut and 60 m into the adjacent forest (Fig. 1). Along each transect we placed six grids of ACOs at 20-m intervals. The edge effect grids consisted of 24 boards (of the same material and dimensions used for harvest effect grids) arranged 3×8 with 3-m spacing between boards. We considered the boundary between forest and harvest (defined by the outermost line of canopy tree trunks) to be 0 m, and we counted distance into the clearcut as negative and distance into the forest as positive. Thus, ACO grids were placed at −40, −20, 0, 20, 40, and 60 m from the edge (Fig. 1).

We sampled edge effect grids during the day (0800 h–1800 h) every two weeks during March–May 2010, September–November 2010, and March–May 2011. Grids were sampled five times during each season. To minimize the effect of time of day, we systematically altered the order in which we checked grids during each sampling occasion. We checked grids by lifting all cover objects and recording the number and species of each salamander encountered.

#### Physical factors

At harvest effect grids, we estimated volume of downed woody debris (DWD) during each spring (2008–2011) using a line-intercept method [43]. At each grid, trained technicians walked two 20-m linear transects, one 5 m upslope and one 5 m downslope of the grid, and recorded the diameter of each piece of DWD ≥10 cm and in contact with the forest floor at the point of intersection. In 2010 and 2011, we also recorded the decay class from 1–5 [44]. Volume (cm^3^/m^2^) was calculated as in Van Wagner [43]. At edge effect grids, we employed the same method, walking four 10-m transects within each grid, during the spring of 2010 and 2011.

To incorporate measures of temperature and moisture into our study, we obtained records of daily precipitation and average daily air temperature from National Atmospheric and Oceanic Administration (NOAA) cooperative stations located around the study area (Martinsville 2SW, ID #125407 for units 1–4; Nashville 2NNE, ID #126056 for units 5–9). At edge effect grids, we also attached Thermochron iButton dataloggers (model DS1921G-F5, Maxim Integrated Products, Sunnyvale, CA) to wooden stakes (10 cm above the ground) located at the upslope edge of each ACO grid and to the underside of one cover object at each grid. We coated dataloggers in red tool dip to waterproof them and set them to record temperature every hour. Although the tool dip coating can affect the temperature recorded by dataloggers, Roznik and Alford [45] find these effects to be relatively small (0–1.3°C). We deployed dataloggers under ACOs beginning 6 March 2010 and on stakes beginning 4 June 2010. Both types then collected data through 12 November 2010 and from 7 March to 5 May 2011.

At edge effect grids we periodically collected additional habitat data including percent canopy cover, leaf litter depth, and soil moisture. We measured canopy cover once each sampling season (in May after leaf out and in September before leaf fall) with a spherical densiometer at five points within each grid (Fig. 1). We measured leaf litter depth once each sampling season (early May and early October) at the same five points with a ruler pressed down to the consolidated soil surface. We averaged these to find a single mean percent canopy cover and single mean litter depth for each grid. We measured percent soil moisture during each sampling occasion using a soil probe (2.54 cm diameter) to collect samples to a depth of 10 cm at the same five points in each grid. In the lab we weighed wet soil samples, placed them in a drying oven for at least five days at 40°C, and weighed them again. We calculated percent soil moisture by subtracting dry weight from wet weight and dividing the difference by the wet weight. We averaged the five samples to find the mean percent soil moisture for each grid on each sampling occasion.

### Statistical analyses

#### Harvest effects

We analyzed the salamander count data in several steps (described in more detail below). We first used two mixed-effects analysis of variance (ANOVA) models to identify significant fixed effects and interactions. Second, we used post-hoc pairwise comparisons to find the direction of significant effects. Third, we used a series of correlation tests to find associations between salamander counts and environmental variables. Finally, we used program PRESENCE (version 4.1, USGS, Laurel, MD) to test a suite of covariates in N-mixture models for estimating abundance while accounting for detection probability. Although some of these steps are partially redundant, no single statistical test could adequately address all relevant research questions in our study.

We analyzed salamander encounters with two mixed-effects ANOVA models. We first standardized the number of sampling occasions per sample period by rarefying the data, removing approximately 10% of the total sampling occasions (those that fell furthest outside the range of sampling dates for a majority of the grids) and leaving 2057 sampling occasions for inclusion in analyses. To circumvent issues with overdispersion, we then summed salamander counts from all sampling occasions within each sample period for each grid, resulting in a single total value for each grid during each sample period. During the fall of 2008 some units were not accessible for sampling due to timber harvest activities, thus the totals and statistics presented herein are from rarefied data that do not include fall 2008. We used one ANOVA model (Model 1) for data from control and group selection grids, in which units were nested in treatment type (i.e., each unit contained only one treatment type). We used a second ANOVA model (Model 2) for data from the remaining four treatment types (clearcut, clearcut adjacent, shelterwood, and shelterwood adjacent), in which treatment type was nested in unit (i.e., each unit contained multiple treatment types). Owing to this difference in nesting, the two models differed in their error structures but their parameterizations were otherwise identical. We tested the fixed effects of treatment type, treatment period (pre- or post- harvest), sample period (a combination of year and season; e.g., fall 2007), slope aspect, and interactions among these terms (specifically, treatment type by treatment period, treatment type by sample period, treatment type by slope aspect, slope aspect by sample period, and the three-way interaction of treatment type by slope aspect by sample period), with volume of DWD as a covariate. In Model 1, we made grid and unit (nested in treatment type) random effects; in Model 2, we made grid and the interaction of unit and treatment type random effects (in both cases, the model statement itself included treatment type as a single fixed effect).

To determine the direction of significant effects and interactions, we used post-hoc Tukey-adjusted pairwise comparison tests of least-square means (LSmeans, also known as marginal means) estimates. To test for differences among the same factors between models, we conducted two-sample *t*-tests on the LSmeans estimates. The additional *t*-tests allowed us to compare indices of relative abundance in control and group selection sites (data from Model 1) to those in clearcut, clearcut adjacent, shelterwood, and shelterwood adjacent sites (data from Model 2). We used a Bonferroni-corrected *α* to account for the multiple *t*-tests.

We conducted Spearman’s rank correlation tests to identify associations between mean salamanders per grid on each sampling day, amount of precipitation during the previous 48 hours, and average daily air temperature. We tested correlations with temperature separately for data from the pre-harvest treatment period, from the post-harvest treatment period at sites where the canopy was retained, and from the post-harvest treatment period at sites where the canopy was removed. Additionally, we conducted Spearman’s rank correlation tests between mean salamanders per grid per sample period and volume of DWD, as well as between salamander counts and volume of DWD in each decay class.

For the ANOVA tests we used square-root transformed count data examined under a Gaussian distribution. The ANOVA tests and the Spearman’s rank correlation tests were conducted for each of the three most commonly encountered species. The *t*-tests comparing means between the two ANOVA models were conducted in Microsoft Excel. All other analyses described above were conducted with SAS software (version 9.2, SAS Institute, Cary, NC). We considered results to be statistically significant at *α* = 0.05.

Estimated abundance based on count data is a function not only of true abundance but also of the observation process [46]. For species such as terrestrial salamanders with cryptic habits, detection is often imperfect, potentially confounding analysis of treatment effects [42], [46]. Thus in addition to our analysis with classical statistics as described above, we used Royle’s [42] N-mixture model for spatially replicated count data in program PRESENCE [47] to estimate abundance *λ* while accounting for detection probability *p* at harvest effect grids. Using raw count data (not rarefied, collapsed, or transformed), we modeled *λ* as a log link function of two site covariates (treatment type and slope aspect) and *p* as a logit link function of one site covariate (average volume of downed woody debris) or three survey covariates (season, precipitation last 48 hrs, and average daily air temperature). Treatment type, slope aspect, and season were all treated as categorical covariates, and DWD, precipitation, and temperature were continuous variables. For each species and treatment period, we first tested the null model with each parameter held constant, *p*(.), λ(.). From this baseline, we held detection probability constant and tested each abundance covariate separately (*p*(.), λ(Cov); two models), and then held abundance constant and tested each detection probability covariate separately (*p*(Cov), *λ*(.); four models). We ranked models by AIC values and used those with the lowest rank to inform combined models which we then tested for improved model performance (Table 1) [48], [49].

**Table 1:** AIC values for N-mixture models for harvest effect grids.

	*P. cinereus*		*P. dorsalis*		*P. glutinosus*	
Model	Pre	Post	Pre	Post	Pre	Post
*p*(.), λ(.)	4545.93	13644.62	3259.44	11949.95	940.58	2680.27
*p*(.), λ(TrtType)	4546.13	13625.33	3260.31	11933.84	941.08	2681.78
*p*(.), λ(Aspect)	4547.16	13643.71	3261.44	11823.91	941.86	2673.30
*p*(Season), λ(.)	4371.30	13599.49	**2761.93**	11739.44	931.05	2681.08
*p*(Precip), λ(.)	4539.63	13643.68	3253.21	11949.95	935.52	2639.44
*p*(Temp), λ(.)	**4207.88**	12777.13	3021.83	11237.96	**912.97**	2615.36
*p*(DWD), λ(.)	4543.80	13573.91	3256.78	11949.65	939.91	2654.45
*p*(Temp), λ(TrtType)	Na	**12764.36**	na	na	na	na
*p*(Temp), λ(Aspect)	Na	na	na	11118.45	na	**2608.66**
*p*(Temp), λ(TrtType+Aspect)	Na	na	na	**11108.54**	na	na

Models were run in program PRESENCE to estimate abundance *λ* and detection probability *p* for repeated count data during the pre-harvest (Pre) and post-harvest (Post) treatment periods at harvest effect grids for eastern red-backed (*Plethodon cinereus*), northern zigzag (*P. dorsalis*), and northern slimy (*P. glutinosus*) salamanders. The lowest AIC values are shown in bold and indicate the best supported model for a given species and treatment period.

#### Edge Effects

We analyzed the effect of distance to edge on salamander relative abundance using a mixed-effects ANOVA model. Again to avoid issues with overdispersion in the data, we collapsed salamander counts across the five visits to each grid within each sampling season (6 grids×6 transects×3 seasons = 108 observations per species) and normalized the collapsed totals with a square-root transformation. We tested the fixed effects of distance to edge, slope aspect, season (spring or fall), and interactions of these terms. We included a random effect of transect nested in study site and a repeated measure of sample season. Following each ANOVA, we conducted post-hoc Tukey-adjusted pairwise comparison tests to identify specific differences in main effects and interactions found to be significant. We repeated this analysis for each species encountered in sufficient numbers to exhibit a normal distribution following transformation.

We conducted Spearman’s rank correlation tests for associations between mean salamanders per grid per sampling occasion, amount of precipitation during the previous 48 hours, average percent soil moisture, air temperature, and temperature under ACOs. We also tested for correlations between mean salamanders per grid per sampling period, volume of DWD, average percent canopy cover, and average leaf litter depth (i.e., habitat characteristics recorded on a seasonal basis). Correlation tests were a necessary alternative to multiple regression due to our inability to normalize the count data without collapsing sampling occasions, which would have precluded use of data collected each sampling occasion (i.e., soil moisture, precipitation, temperature). The remaining variables (i.e., DWD, canopy cover, leaf litter) were also better examined through correlation tests rather than as part of the ANOVA models due to strong correlations among these and distance to edge. We plotted physical factors over distance to edge to visually demonstrate how they fluctuated across the edge gradient. The ANOVA and correlation analyses for edge effects were conducted in SAS, and we considered results significant at *α* = 0.05.

As with harvest effect data, we used N-mixture models in program PRESENCE to determine the best covariates to estimate abundance *λ* and detection probability *p* from repeated counts at edge effect grids. For each species we tested two site covariates (distance to edge and slope aspect) for *λ* and three site covariates (average volume of DWD per grid, average percent canopy cover, average depth of leaf litter) or four survey covariates (season, precipitation last 48 hrs, ACO temperature, and average percent soil moisture) for detection probability. We again tested the null model and each covariate separately. A final set of combined models were ranked according to AIC values (Table 2).

**Table 2:** AIC values for N-mixture models for edge effect grids.

Model	*P. cinereus*	*P. dorsalis*	*P. glutinosus*
*p*(.), λ(.)	2560.41	3101.52	597.82
*p*(.), λ(Distance)	2547.33	3103.34	599.69
*p*(.), λ(Aspect)	2549.50	3103.52	588.05
*p*(Season), λ(.)	2368.16	2863.18	592.99
*p*(Precip), λ(.)	2551.70	3092.50	599.65
*p*(ACOTemp), λ(.)	2562.34	3100.94	597.10
*p*(DWD), λ(.)	2561.42	3100.82	599.61
*p*(Soil), λ(.)	2405.31	2879.68	584.85
*p*(AvgCanopy), λ(.)	2543.43	3099.96	595.93
*p*(AvgLitter), λ(.)	2548.95	3080.67	595.72
*p*(Season), λ(Distance+Aspect)	**2345.60**	na	na
*p*(Season+Soil), λ(Distance+Aspect)	2345.96	na	na
*p*(Season+Soil), λ(.)	na	**2860.99**	na
*p*(Soil), λ(Aspect)	na	na	**577.22**

Models were run in program PRESENCE to estimate abundance *λ* and detection probability *p* for repeated count data from edge effect grids for eastern red-backed (*Plethodon cinereus*), northern zigzag (*P. dorsalis*), and northern slimy (*P. glutinosus*) salamanders. The lowest AIC values are shown in bold and indicate the best supported model for a given species.

## Results

### Harvest Effects

We recorded 19,048 salamander encounters (nine species) under ACOs at harvest effect grids from fall 2007 to spring 2011 (S2 Table). Eastern red-backed salamanders (*Plethodon cinereus*, n = 11,259), northern zigzag salamanders (*P. dorsalis,* n = 6913), and northern slimy salamanders (*P. glutinosus*, n = 752) accounted for 99% of all salamander encounters. Six salamander species were encountered in insufficient numbers (<1% of total encounters) to be included in analyses. Though we did not statistically analyze encounters of non-target species, reptile encounters were greatest in the post-harvest period and concentrated in sites with timber removal (S2 Table).

The following section includes results for the ANOVA models, which included simple fixed effects as well as interaction terms as described previously. Although all of these terms were important to include in the model to account for their effect on the data, not all significant fixed effects provide useful information about salamander abundance. For example, although treatment type may be a significant fixed effect, a comparison of the means of each treatment type would be confused by the inclusion of both pre- and post-harvest data. In this case, the interaction of treatment type and treatment period would provide better insight into the effects of both variables on salamander counts. Therefore, the results described below include post-hoc tests only for those fixed effects and interaction terms that address research questions of interest.

#### Plethodon cinereus

**Figure 2:**
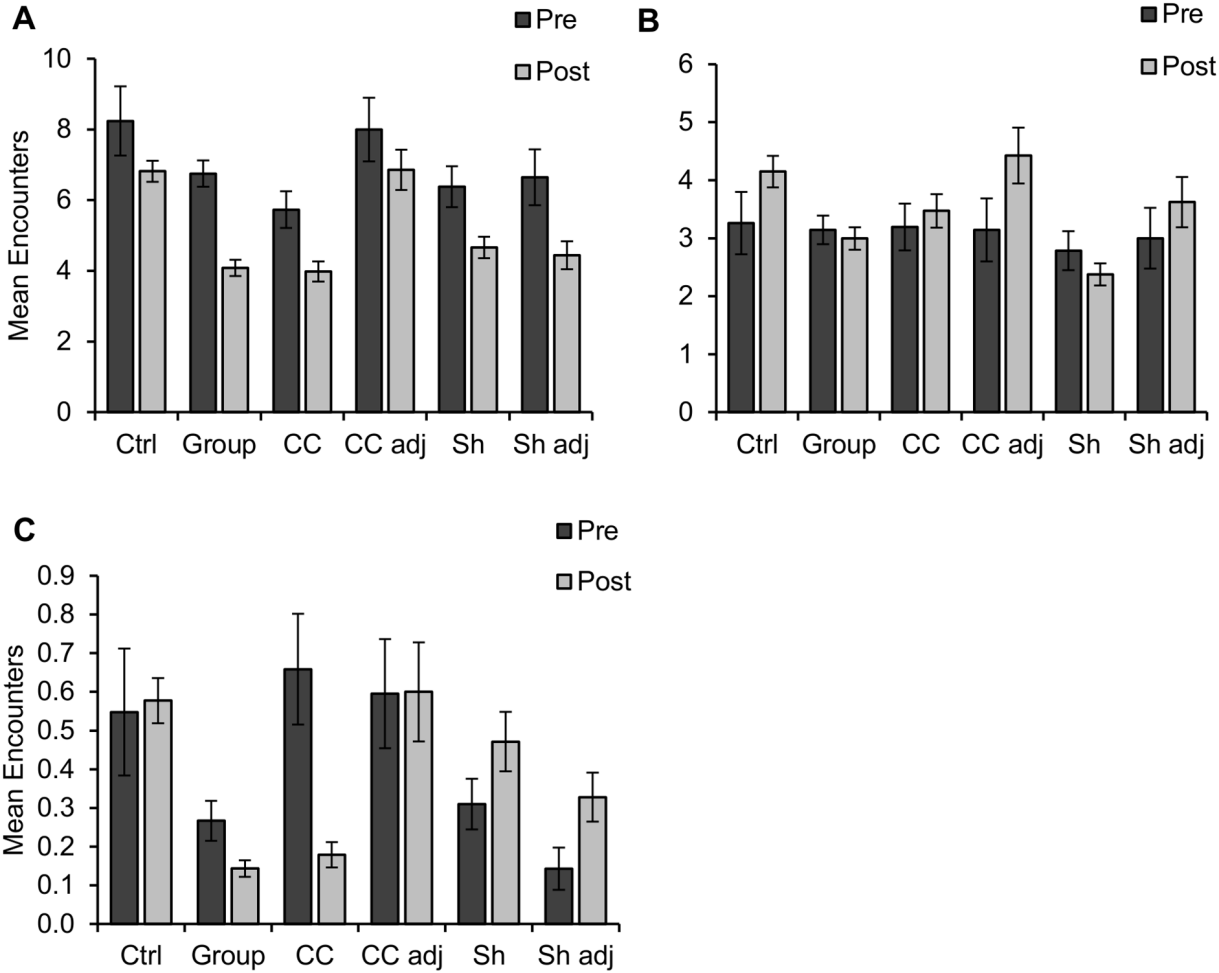
Salamander encounters by treatment type and treatment period. Mean encounters per sampling occasion decreased significantly from pre- (fall 2007 and spring 2008) to post-harvest (spring and fall 2009, 2010, and spring 2011) in group cuts and clearcuts for (A) eastern red-backed salamanders (*Plethodon cinereus*), and (C) slimy salamanders (*P. glutinosus*). Mean encounters of red-backed salamanders also decreased significantly in control sites, while mean encounters of (B) northern zigzag salamanders (*P. dorsalis*) increased significantly in clearcut adjacent sites. One sampling occasion consists of a single visit to a single cover-board grid. Error bars represent ± standard error. Results were considered significant at *α* = 0.05. Ctrl = control; Group = group selection; CC = clearcut; CC adj = clearcut adjacent; Sh = shelterwood; Sh adj = shelterwood adjacent.

Treatment type, treatment period, sample period, the treatment type by sample period interaction and the slope aspect by sample period interaction were significant effects in ANOVA Model 1 for red-backed salamanders (S3 Table). These same effects, excepting the treatment type by sample period interaction, were also significant in Model 2 (S4 Table). Using post-hoc tests to determine the direction of these effects, we found that mean encounters of red-backed salamanders decreased from pre- to post-harvest in control (*p = *0.025), group (*p<*0.001), and clearcut sites (*p<*0.001; Fig. 2a). There were no differences among treatment types during the pre-harvest treatment period, but during the post-harvest treatment period control sites had greater encounters than group cuts (*p* = 0.004), and clearcut adjacent sites had greater encounters than clearcuts (*p* = 0.031).

We examined pairwise differences in salamander encounters among treatment types within each sample period, first regardless of slope aspects (slope aspects pooled), and then again with each slope aspect category (northeast and southwest) considered separately (slope aspects not pooled). With slope aspects pooled, encounters of red-backed salamanders were greater in control sites than in group cuts during fall 2010 (*p<*0.001, S1 Figure). The same was true when we examined northeast-facing slopes alone (*p<*0.001). Southwest-facing slopes did not show any differences among treatment types within any sample period pre- or post-harvest.

#### Plethodon dorsalis

Sample period, slope aspect, treatment type by sample period, treatment type by slope aspect, slope aspect by sample period, and volume of DWD were significant effects on zigzag salamanders in ANOVA Model 1 (S3 Table). In Model 2, treatment type, treatment period, sample period, slope aspect, treatment type by treatment period, and treatment type by slope aspect were significant (S4 Table). Encounters were greater on northeast-facing slopes than on southwest-facing slopes in Models 1 (*p* = 0.001) and 2 (*p*<0.001). Clearcut adjacent sites increased in mean zigzag salamander encounters from pre- to post-harvest (*p* = 0.038; Fig. 2b). Treatment types did not differ pre-harvest, but during the post-harvest treatment period zigzag salamander encounters were greater on clearcut adjacent sites than in shelterwoods (*p* = 0.012). We found no differences in mean zigzag salamander encounters among treatment types in any sample period with slope aspects pooled (S1 Figure). On northeast-facing slopes, encounters were greater in controls compared to group cuts (*p* = 0.006) and shelterwoods (*p*<0.001) in fall 2010.

#### Plethodon glutinosus

Treatment period, sample period, slope aspect, and volume of DWD were significant effects on slimy salamanders in ANOVA Model 1 (S3 Table), while treatment type, sample period, slope aspect, and treatment type by treatment period were significant in Model 2 (S4 Table). Mean encounters of slimy salamanders decreased from pre- to post-harvest in group cuts (*p<*0.001), and clearcuts (*p<*0.001; Fig. 2c). During the post-harvest treatment period, slimy salamander counts were lower in clearcuts than in shelterwoods (*p* = 0.013), clearcut adjacent sites (*p = *0.002), and shelterwood adjacent sites (*p = *0.020). Encounters were greater on northeast-facing slopes than on southwest-facing slopes (*p*<0.01). With slope aspects pooled, mean encounters of slimy salamanders were greater in shelterwood adjacent sites than in clearcut sites in fall 2010 (*p = *0.029; S1 Figure).

#### Physical factors

Average daily air temperature was significantly and negatively correlated with counts of each species during the pre-harvest period and during the post-harvest period, whether the canopy was retained or removed (Table 3). Total DWD and DWD in decay class 2 were negatively correlated with counts of red-backed salamanders. Counts of zigzag salamanders were positively correlated with DWD in decay class 1, while counts of slimy salamanders were negatively correlated with DWD in decay classes 2 and 3 but positively correlated with DWD in decay class 5 (Table 3).

**Table 3:** Spearman rank correlation tests for associations between salamander encounters and environmental variables at harvest effect grids.

		*P. cinereus*		*P. dorsalis*		*P. glutinosus*	
Physical factor	n	r_s_	*P*	r_s_	*p*	r_s_	*p*
Precipitation^a^	232	−0.03	0.681	0.02	0.757	0.04	0.504
Temp, pre^b^	61	−0.36	<0.001*	−0.39	<0.001*	0.24	0.022*
Temp, post, retained^c^	126	−0.41	<0.001*	−0.38	<0.001*	0.26	0.003*
Temp, post, removed^d^	129	−0.56	<0.001*	−0.50	<0.001*	0.26	0.003*
DWD^e^	527	−0.17	<0.001*	−0.08	0.071	−0.08	0.055
DWD decay 1^f^	250	−0.04	0.489	0.15	0.019*	−0.08	0.183
DWD decay 2	250	−0.27	<0.001*	−0.06	0.324	−0.14	0.024*
DWD decay 3	250	0.03	0.584	0.09	0.165	−0.17	0.006*
DWD decay 4	250	0.03	0.689	−0.01	0.849	0.06	0.308
DWD decay 5	250	0.05	0.466	−0.10	0.115	0.21	<0.001*

Tests were conducted for counts of eastern red-backed (*Plethodon cinereus*), northern zigzag (*P. dorsalis*), and northern slimy (*P. glutinosus*) salamanders.

*Significant effect at *α* = 0.05.

a Precipitation 48 hours prior to sampling vs. mean salamanders per grid per sampling day (treatment types and sample periods pooled).

b Average daily air temperature vs. mean salamanders per sampling occasion during the pre-harvest period (treatment types pooled).

c Average daily air temperature vs. mean salamanders per sampling occasion during the post-harvest period where canopy was retained (control, clearcut adjacent, shelterwood, and shelterwood adjacent).

d Average daily air temperature vs. mean salamanders per sampling occasion during the post-harvest period where canopy was removed (clearcuts and group cuts).

e Volume of downed woody debris (all decay classes) at each grid vs. mean salamanders per grid per sample period (treatment types pooled).

f DWD by decay class (1 = little decayed; 5 = well decayed [44]); data from 2010 and 2011 only.

#### N-mixture models

During the pre-harvest period, abundance for each species was better modeled as constant than as varying by treatment type or slope aspect (Table 1). During the post-harvest period, abundance was best modeled as varying by treatment type for red-backed salamanders, by treatment type and slope aspect for zigzag salamanders, and by slope aspect for slimy salamanders. As mentioned above, counts of zigzag and slimy salamanders were greater on northeast-facing slopes than on southwest-facing slopes. Detection probability was best modeled by average daily air temperature for each species during both the pre- and post-harvest periods, with the exception of zigzag salamanders during the pre-harvest period, where detection probability was modeled best by season. Salamander counts for all species were negatively correlated with average daily air temperature in Spearman correlation tests. Mean encounters of zigzag salamanders during the pre-harvest treatment period were greater in the spring (16.0±0.6) than the fall (5.4±1.0). For post-harvest data, models that combined the most favored covariate for abundance and detection probability for a given species outcompeted models with a single covariate (Table 1).

### Edge Effects

From 2010 to 2011 we observed 2727 salamander encounters under ACOs at the six edge transects (S5 Table). Captures included seven salamander species, primarily composed of zigzag salamanders (n = 1442; 53% of encounters) and red-backed salamanders (n = 1137; 42% of encounters). We were able to normalize encounters of these two species; slimy salamanders made up 4% of salamander encounters and could not be normalized, but we included this species in non-parametric correlation tests and in N-mixture model analyses.

#### Plethodon cinereus

**Figure 3:**
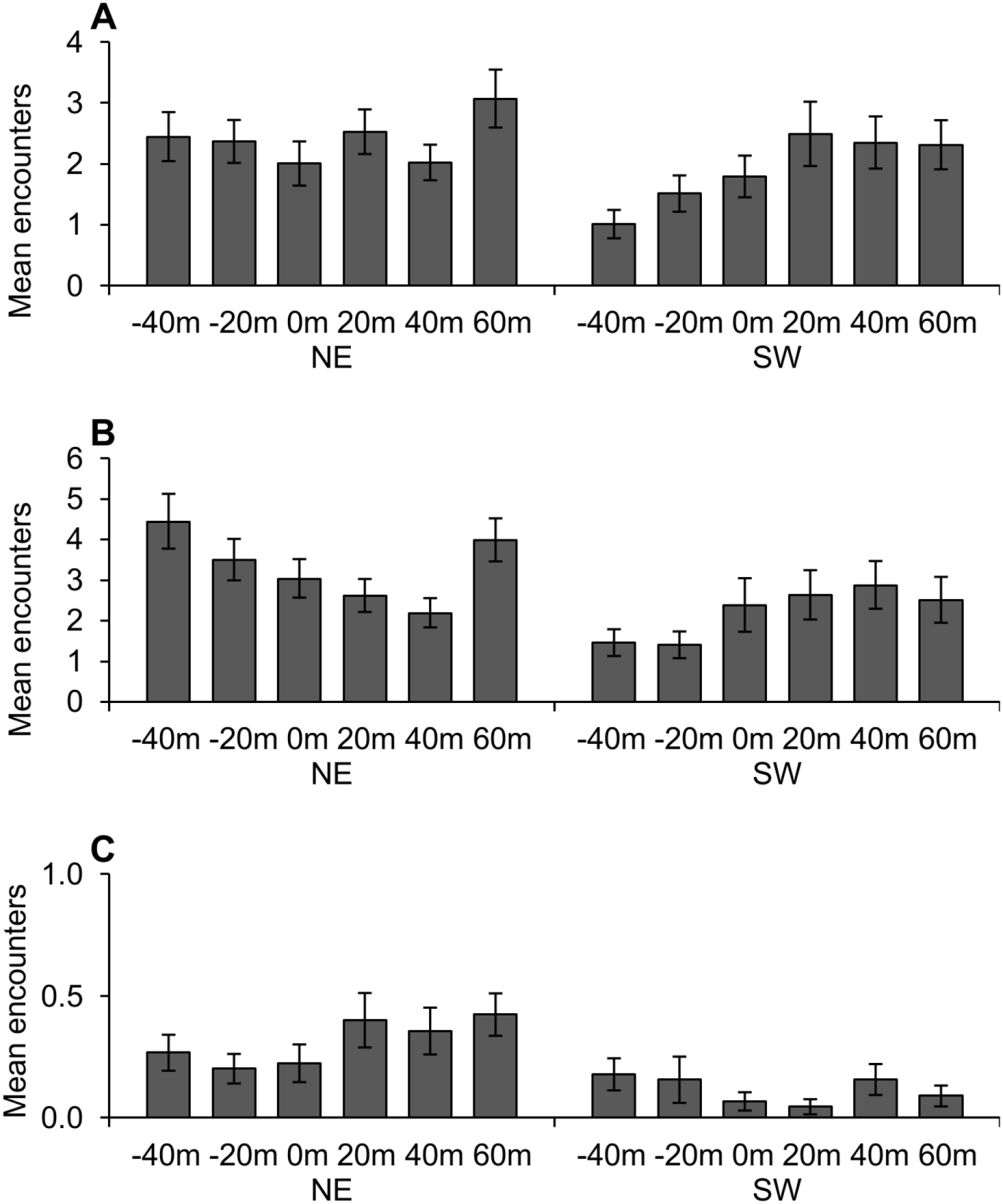
Salamander encounters by distance to edge and by slope aspect. Mean encounters per sampling occasion of (A) eastern red-backed salamanders (*Plethodon cinereus*) at edge effect grids from March 2010 to March 2011 were significantly greater at 20, 40, and 60 m than at −40 m on southwest slopes (SW, n = 3). Mean encounters of (B) zigzag salamanders (*P. dorsalis*) were significantly greater at −40 m than at 40 m on northeast slopes (NE, n = 3). Encounters of both species generally increased from the clearcut interior to the forest interior on southwest slopes. Counts of (C) slimy salamanders (*P. glutinosus*) are presented graphically but were low and could not be normalized for analysis. Error bars represent ± standard error. Results were considered significant at *α* = 0.05.

The relative abundance of red-backed salamanders was significantly affected by all tested factors and interactions excepting slope aspect (Table 4). Mean counts were greater in the spring than the fall (*p<*0.001), and at 60 m inside the forest than at 0 m (*p = *0.003) or at −40 m (*p = *0.006). From post-hoc tests of the distance by slope aspect interaction we found that on southwest-facing slopes, mean encounters were greater inside the forest at 20, 40, and 60 m than inside the clearcut at −40 m (*p = *0.036, *p = *0.006, and *p = *0.005, respectively; Fig. 3a). During the spring, mean counts were greater at 60 m than at either clearcut interior distance (−20 m, *p* = 0.031; −40 m, *p = *0.003), and mean counts were greater at 20 m than at −40 m (*p* = 0.008); during the fall, mean counts were greater at both 60 m and −20 m than at 0 m (*p = *0.036 and *p = *0.044, respectively).

**Table 4:** Type III fixed effects for analysis of variance of salamander counts at edge effect grids.

	*P. cinereus*		*P. dorsalis*	
Effect^a^	F	*p*	F	*p*
Dist^b^	4.24	0.002*	0.79	0.557
A^c^	1.73	0.259	1.72	0.260
Dist×A	3.54	0.006*	4.50	0.001*
Season^d^	38.00	<0.001*	18.68	0.001*
Dist×Season	4.02	0.003*	1.23	0.303

Models were run for eastern red-backed (*Plethodon cinereus*) and northern zigzag (*P. dorsalis*) salamanders.

*Significant effect at *α* = 0.05.

a Interaction terms are indicated by an ‘×’ between two or more factors.

b Dist = distance to edge.

c A = slope aspect (northeast or southwest).

d fall or spring in a given year.

#### Plethodon dorsalis

The relative abundance of zigzag salamanders was significantly affected by season and the interaction of distance and slope aspect (Table 4). Mean counts were greater in the spring than the fall (*p = *0.001). On northeast-facing slopes alone, mean counts were greater at −40 m than at 40 m (*p = *0.032; Fig. 3b); on southwest-facing slopes, mean counts generally increased with distance from the clearcut interior into the forest, but we found no other differences in pairwise combinations of distance and slope aspect for zigzag salamanders.

#### Physical factors

**Figure 4:**
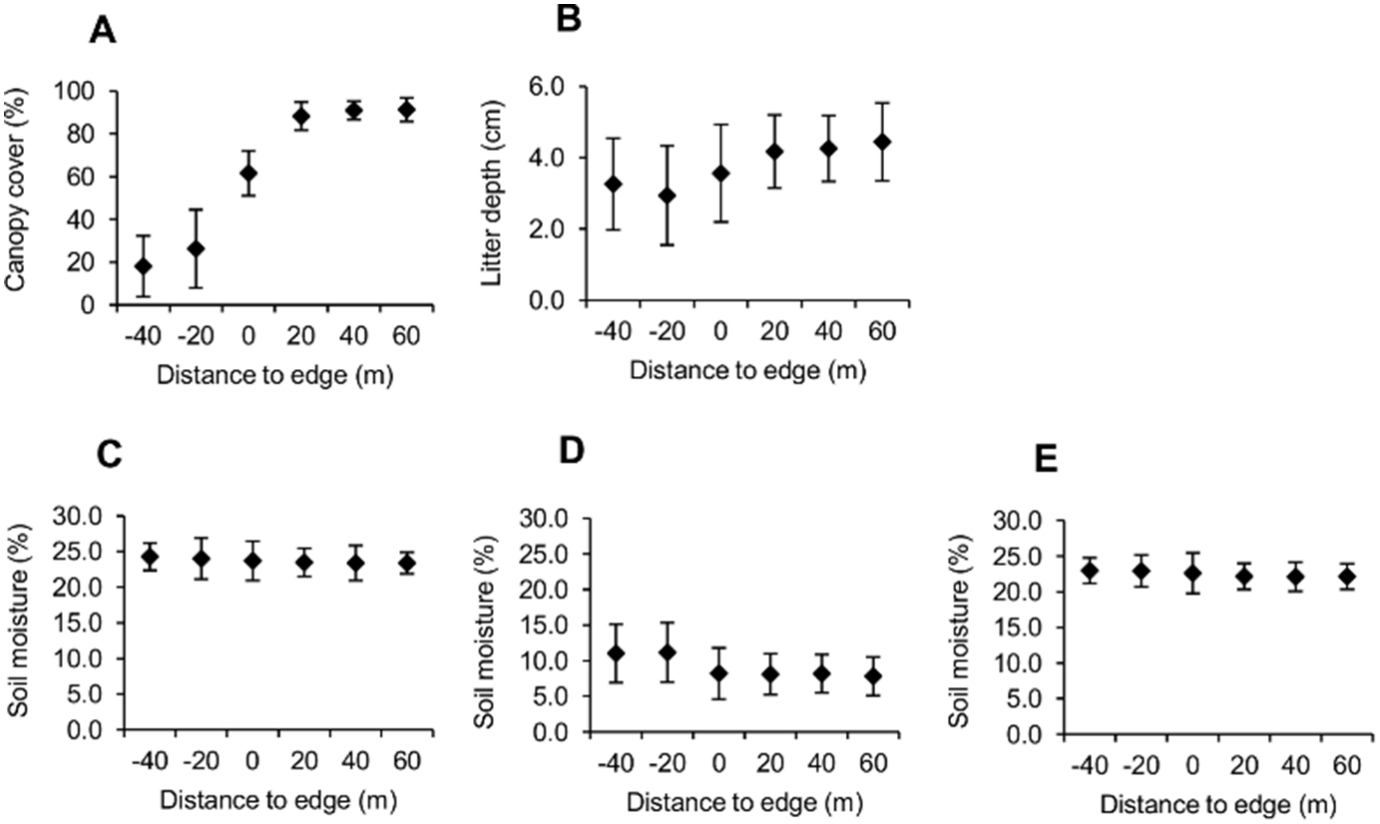
Habitat characteristics (mean ± 1SE) across the edge gradient. Canopy cover, leaf litter depth, and soil moisture were measured at five points within each grid of each edge transect (n = 6 grids×6 transects = 36). (A) Percent canopy cover and (B) litter depth increased with distance away from the edge into the forest, while percent soil moisture was fairly constant across distance intervals but varied greatly by season, being much greater during (C) spring 2010 and (E) spring 2011 than during (D) fall 2010.

Percent canopy cover and depth of leaf litter were low at clearcut interior distances and increased with proximity to the forest interior (Fig. 4). Soil moisture did not vary greatly across the edge gradient but was much greater during spring sample periods than during the fall of 2010 (Fig. 4). Correlations between salamander counts and physical factors at edge effect grids varied by species (Table 5). Counts of red-backed and zigzag salamanders at edge effect grids were positively correlated with recent precipitation and negatively correlated with temperature under ACOs; counts of slimy salamanders were positively correlated with temperature under ACOs. All three species were positively correlated with percent soil moisture, but only red-backed salamanders were positively correlated with percent canopy cover, and only zigzag and slimy salamanders exhibited positive correlations with DWD in older decay classes (Table 5).

**Table 5:** Spearman rank correlation tests for associations between salamander encounters and environmental variables at edge effect grids.

		*P. cinereus*		*P. dorsalis*		*P. glutinosus*	
Physical factor	N	r_s_	*P*	r_s_	*P*	r_s_	*p*
Precipitation^a^	45	0.19	0.216	0.10	0.529	−0.02	0.888
AirTemp^b^	353	−0.05	0.323	−0.01	0.838	−0.01	0.877
ACOTemp^c^	518	−0.37	<0.001*	−0.33	<0.001*	0.10	0.023*
Soil^d^	539	0.28	<0.001*	0.32	<0.001*	0.17	<0.001*
Canopy^e^	108	0.27	0.005*	0.12	0.264	0.13	0.177
Litter^f^	108	0.09	0.344	0.18	0.064	0.00	0.986
DWD^g^	108	−0.03	0.749	0.17	0.842	0.05	0.612
DWD decay 1^h^	108	0.03	0.721	−0.06	0.842	−0.11	0.236
DWD decay 2	108	−0.16	0.093	−0.06	0.546	−0.13	0.194
DWD decay 3	108	0.13	0.197	0.26	0.007*	0.16	0.106
DWD decay 4	108	0.04	0.703	0.22	0.022*	0.13	0.187
DWD decay 5	108	0.07	0.450	0.23	0.016*	0.34	<0.001*

Tests were conducted for counts of eastern red-backed (*Plethodon cinereus*), northern zigzag (*P. dorsalis*), and northern slimy (*P. glutinosus*) salamanders.

*Significant effect at *α* = 0.05.

a Precipitation 48 hours prior to sampling vs. mean salamanders per grid per sampling day.

b Air temperature as recorded by data loggers on stakes at ACO grids vs. salamander count per sampling occasion.

c Temperature as recorded by data loggers under ACOs vs. salamander count per sampling occasion.

d Average percent soil moisture vs. salamander count per sampling occasion.

e Average percent canopy cover vs. mean salamanders per grid per sample period.

f Average depth of leaf litter vs. mean salamanders per grid per sample period.

g Volume of downed woody debris (all decay classes) vs. mean salamanders per grid per sample period.

h DWD by decay class (1 = little decayed; 5 = well decayed [44]).

#### N-mixture models

N-mixture models found that abundance at edge effect grids was best modeled by distance and slope aspect for red-backed salamanders, as constant for zigzag salamanders, and by slope aspect for slimy salamanders (Table 2). As previously described and as seen in Fig. 3, mean encounters of all species were generally higher on northeast-facing slopes than on southwest-facing slopes, and on southwest-facing slopes mean encounters of red-backed and zigzag salamanders generally increased from the clearcut interior to 20 m inside the forest. Detection probability was best modeled by season for red-backed salamanders, by season and soil moisture for zigzag salamanders, and by soil moisture for slimy salamanders. Mean encounters of red-backed and zigzag salamanders were greater in the spring (13.3±0.8 and 12.3±1.5, respectively) than the fall (4.8±0.7 and 6.4±1.3, respectively), and counts of zigzag and slimy salamanders were positively associated with percent soil moisture (Table 5). The lowest AIC ranks were found for models containing combinations of these covariates (Table 2).

## Discussion

### Harvest Effects

We found clearcuts and group cuts had a negative impact on the local Plethodontid salamander community during the first three years following harvest. Indeed, we detected post-harvest differences among treatment types which were not evident during the pre-harvest treatment period. The direction of our results for clearcuts are reasonably consistent with those reviewed by deMaynadier & Hunter [21], where median captures of salamanders were found to be 4.3 times greater in control stands than in clearcut stands; however, the magnitude of this difference was less in our study, with post-harvest salamander counts only about 1–3 times higher in controls than in clearcuts (1.7 for red-backed salamanders, 1.2 for zigzag salamanders, and 3.2 for slimy salamanders, as calculated from S6 Table). The HEE was designed to reflect the dominant forest management strategies of the region, and thus relatively small clearcuts (4 ha), shelterwoods (4 ha), and group cuts (0.4–2 ha; also termed patch cuts) were installed. Clearcuts investigated in other studies are often larger than 4 ha [50]–[52]. Smaller harvest areas may have less extreme effects on salamanders because changes in factors such as solar radiation and wind exposure are less extreme than they are in larger harvests, as suggested by differences in tree species composition among 30-year-old clearcuts of varying sizes [53]. The moderate effects we found fall closer to the results of Renken et al. [25], in which differences in the abundance of most of 13 amphibian and reptile species up to three years post-harvest were not detected at the landscape scale. Like the present study, this research took place in the Midwestern US, under a study design similar to that of the HEE (i.e., size, replication, and method of harvest).

The effects of group selection harvesting on salamanders are less well-studied than those of clearcuts, but some studies suggest small, selective cuts have little to no effect on salamander abundance [25], [31], or that negative effects are less severe than those in clearcuts [30]. Our results show group cuts have a negative effect similar to that of clearcuts, although these effects were not evident for all species. The long-term effects of group selection could be more detrimental than those of clearcutting, given the need to harvest across a larger area or harvest at more frequent intervals to produce the same amount of timber [29], [30].

One seemingly confounding result is the significant decline of red-backed salamanders in control sites between the pre- and post-harvest periods, mirroring that seen in group cuts and clearcuts (Fig. 2a). This suggests factors other than the harvests influenced declines in salamander relative abundance. However, declines at the treated sites were steeper than those seen at control sites. Despite declines from pre- to post-harvest, control sites still had significantly greater mean encounters of red-backed salamanders during the post-harvest period than did group cuts. Mean encounters on control sites were also greater during the post-harvest period that those in clearcuts, although this difference was not found to be significant, probably due to the conservative Bonferroni-corrected *α* used in post-hoc comparisons between ANOVA Models 1 and 2. Thus, although factors other than the treatments must have been involved in salamander declines, treatments were likely at least partially responsible for observed declines at clearcuts and group cuts.

In direct contrast to the salamander response observed in clearcuts and group cuts, salamander relative abundance was not generally affected by the understory treatment (i.e., first stage) of a three-stage shelterwood harvest. Other studies have found reduced salamander relative abundance in shelterwood harvests immediately following the first stage harvest [29], [54], [55], however, at least one study found evidence that these effects are only short-lived (<5 years) [55]. The largely intact canopy of the shelterwood prepatory cut is the most obvious difference that may account for the limited impact of this treatment compared to clearcuts and group cuts. As with group cuts, long-term monitoring is needed to understand the effects of future stand entry and timber removal with this harvest technique.

The results of the N-mixture model analyses lend support to our conclusions about harvest effects. Since the N-mixture models considered each treatment period separately and tested treatment type as a covariate of abundance, together the models essentially incorporated the interaction term of treatment type by treatment period. In agreement with the ANOVA results showing a lack of treatment type differences during the pre-harvest period, the N-mixture models did not favor inclusion of treatment type as a covariate for abundance during the pre-harvest period (Table 1). Treatment type was included in post-harvest models for red-backed and zigzag salamanders, although it was not an important covariate for slimy salamanders, which were primarily influenced by slope aspect (Table 1). These results suggest that even accounting for detection probability, salamander abundance was likely influenced by timber harvests. The N-mixture model result that slope aspect was an important covariate for abundance for zigzag and slimy salamanders (but not red-backed salamanders) follows the ANOVA result that slope aspect was significant for these two species (and not red-backed salamanders), with greater encounters on northeast-facing slopes than on southwest-facing slopes.

An important finding of this study is the consistent effect of sample period across all species at harvest effect grids (Tables 4 and 5). This temporal variation was largely independent of the timber harvests, as treatment type and sample period only interacted for some species and models. Other studies of terrestrial salamanders have also found seasonal and yearly variation to be important factors influencing abundance [25], [33]. Year and seasonal differences are likely driven primarily by patterns of precipitation and temperature [33], as exemplified by the concentration of pairwise differences among treatment types during the drought season of fall 2010 [56]. This is also supported by the finding of the N-mixture models that temperature was an important covariate for detection probability.

### Edge Effects

Few studies have examined the effects of silvicultural edges on terrestrial salamanders. In Maine, deMaynadier and Hunter [32] estimated the depth of edge influence (DEI) of recent clearcuts on red-backed salamanders to be 25–35 m. In New Hampshire, DeGraaf and Yamasaki [33] observed low counts of red-backed salamanders in regenerating stands, increased abundance at the edge and peak abundance 20 m into the forest, suggesting a DEI of 20 m or less [33]. We observed a similar trend on southwest-facing slopes but this trend was either not evident (red-backed salamanders) or reversed (zigzag salamanders) on northeast-facing slopes (Fig. 3). The N-mixture analysis supported that distance to edge and slope aspect were important factors influencing red-backed salamander abundance, and that slope aspect was an important influence on slimy salamander abundance (Table 2). The mechanisms behind the edge effects we observed are likely related to physical factors and habitat characteristics that also varied across the edge gradient and by slope aspect, which we discuss in the following section along with results from harvest effect grids.

### Physical factors

Due to unequal exposure to solar energy [7], northeast-facing slopes in the northern hemisphere are typically characterized by relatively cooler and wetter conditions favored by salamanders, while southwest-facing slopes are more often characterized by hotter and drier conditions [6]. Throughout our study salamander counts were consistently higher on northeast-facing slopes than on southwest-facing slopes, and edge effects on salamanders were stronger on southwest-facing slopes (Fig. 3). This is not unexpected, since the intensity of physical edge effects is also known to vary by slope orientation [5]. In the context of forest management, these differences suggest conducting timber harvests on northeast-facing slopes could potentially mitigate negative effects on local salamander populations. However, we observed few interactions between slope aspect, treatment type, and sample period to support this theory, and such a strategy could also mean disturbances would be concentrated in the highest-quality habitat. Thus, the difference in moisture regimes among slope aspects is likely not enough to completely mitigate harvest effects on salamanders.

Salamander counts were not associated with precipitation in this study (Table 3), though such a relationship has been observed elsewhere [18]. Soil moisture, which may be a more accurate measurement of microhabitat moisture conditions, was positively correlated with salamander counts at edge effect grids (Table 5) and was included as a covariate of detection probability in top N-mixture models of salamander counts at edge transects (Table 2). This agrees with other studies that suggest soil conditions are important in determining salamander abundance and distribution [31], [33], [57], [58]. While average percent soil moisture did not appear to vary across distance intervals at the edge gradient, it was much greater in either spring season than during the fall of 2010. This was a period of drought in southern Indiana and also coincided with particularly low salamander counts at nearly all sites, further suggesting soil moisture is a key factor in salamander detection under ACOs.

The strongest and most consistent correlation we found among physical factors and salamander counts was that of temperature. Average daily air temperature and temperature under ACOs was negatively correlated with counts of red-backed and zigzag salamanders, and positively correlated with counts of slimy salamanders (Table 3). The difference in species response to temperature is likely due to the larger adult size reached by slimy salamanders (total length 11.5–20.5 cm), which affords them a lower rate of dehydration [59] than the smaller red-backed and zigzag salamanders (total length 6.5–12.5 cm; [19]). Temperature was also identified as an important covariate of detection by N-mixture models for harvest effect data (Table 1). The permeable skin of Plethodontid salamanders that allows for cutaneous respiration not only requires moisture but provides a limited defense against desiccation. It is therefore unsurprising that small species should retreat from the surface to avoid warmer temperatures. Our results indicate future studies of salamanders and forest disturbance should account for variation caused by temperature.

Salamander counts along the edge gradient increased with increasing percent canopy cover (red-backed salamanders) but not leaf litter depth (Table 5). Litter depth followed the same trend as canopy cover across distance intervals, but to a lesser extreme (Fig. 4). A similar relationship with canopy cover was seen with red-backed salamanders across an edge gradient in Maine, though the same study also found a positive relationship with litter depth [32]. Conversely, a study in Georgia found no significant correlation between salamander species diversity, species richness, or measures of relative abundance with leaf litter depth or loose soil depth [60]. Shade provided by the canopy plays a direct role in temperature regimes, which we also observed having a strong effect on salamander counts under ACOs. Terrestrial salamanders forage in leaf litter and litter depth undoubtedly contributes to habitat quality for salamanders, but we did not find it to be a particularly important factor on its own.

Volume of downed woody debris has the potential to influence salamander relative abundance [21], [55] and detection rates of ACOs [61]. High volume of DWD may predispose a site to high salamander abundance by providing suitable microhabitat. Alternatively, abundant natural cover may cause salamanders not to use artificial cover as readily as on sites where natural woody debris is sparse. We found only limited effects of DWD on salamander counts. The negative correlation between red-backed salamander counts at harvest effect grids and total volume of DWD had a small correlation coefficient (Table 3). Other correlations generally found counts to be negatively correlated with DWD in lower decay classes or positively correlated with DWD in more advanced decay classes (Table 3), likely reflecting the greater moisture retention and available refugia within debris in advanced stages of decay. It is possible we did not see a strong effect of DWD due to a limited range of volumes across study sites (0.00 to 288.40 m^3^/ha). Our method of sampling DWD was not as intensive as methods outlined elsewhere, such as those used in studies of forest fuel loads (e.g., [62]), and we only recorded decay class during the last two years of sampling. Thus, our finding of only limited effects of DWD on salamander counts should not be taken as clear evidence that woody material is unimportant to terrestrial salamanders, as several studies have found it to be a significant habitat characteristic [23], [30], [50], [63].

### Conclusions

Harvest treatments that removed the forest canopy had moderate negative effects on the relative abundance of terrestrial salamanders, at least at the local scale. Techniques that left the forest canopy intact did not result in salamander declines. Our results demonstrate the importance of analyzing individual species separately, as effects differed even among species within the same genus (Fig. 2). Our data also suggest it is important to monitor salamanders over multiple seasons and years for as long as is feasible, since temporal variation may lead to erroneous conclusions from shorter-term studies. Temperature, soil moisture, and canopy cover were important correlates of salamander relative abundance. We observed edge effects of recent clearcuts on salamanders extending only 20 m or less into the forest, and only on southwest-facing slopes. This depth of edge influence is similar to the 25–35 m DEI estimated by other studies conducted in the eastern US of terrestrial salamanders at silvicultural edges [32], [33].

The precise fate of salamanders in harvested sites remains difficult to ascertain. Artificial cover objects provide only an index of salamander abundance from the limited proportion of the population present at the soil surface [64]. Although we observed reductions in salamander counts of most species in clearcut and group cut sites, we could not determine whether salamanders perished on site, relocated to adjacent forest, or retreated to underground burrows [26], [65]. The increase in zigzag salamander counts in sites adjacent to clearcuts following harvest (Fig. 2b) suggests individuals may have moved from clearcuts to adjacent forested sites, but we did not observe a significant decline of zigzag salamanders in clearcut sites following harvest, nor did we mark individuals to track their movements. Such time-consuming and labor-intensive methods require a compromise of scale of study, but nevertheless provide valuable information and should be pursued when feasible.

The harvest effects we observed were limited to the site of harvest and we found no indication of wider effects on salamander abundance across the landscape. The relatively small harvest openings used in the HEE may therefore be compatible with maintaining terrestrial salamander populations in a contiguous hardwood forest landscape. Although multiple small harvests result in a greater prevalence of edge habitat, our finding of a relatively small depth of edge influence suggests this could be a viable strategy for salamanders. We encourage further study of the fate of salamanders at harvest sites so we may fully understand mechanisms of local declines. Given worldwide amphibian declines [66], [67] and the value of salamanders to forest ecosystems [15], [20], it remains important to monitor the impacts of disturbance on salamanders and strive to adjust forest management practices to best encompass both social and ecological needs.

## Supporting Information

S1 Figure **Mean encounters by treatment type and sample period.** Mean encounters of red-backed (*Plethodon cinereus*, REBA), zigzag (*P. dorsalis*, ZIZA), and northern slimy (*P. glutinosus*, NOSL) salamanders per sampling occasion by sample period at (A) control, (B) group selection, (C), clearcut, (D) clearcut adjacent, (E) shelterwood and (F) shelterwood adjacent treatment sites. Means are calculated from rarefied data. Error bars represent ± standard error.
(TIF)

S1 Table **Published studies on salamanders and timber harvests.** Studies investigating the effects of silvicultural treatments on terrestrial salamander abundance in North America.
(DOCX)

S2 Table **Total encounters at harvest effect grids**. Total encounters of amphibians and reptiles by treatment type and treatment period.
(DOCX)

S3 Table **Type III fixed effects for analysis of variance Model 1 (data from control sites and group cuts).** Asterisks indicate significant effects at *α* = 0.05.
(DOCX)

S4 Table **Type III fixed effects for analysis of variance Model 2 (data from clearcut, clearcut adjacent, shelterwood, and shelterwood adjacent treatment types).** Asterisks indicate significant effects at *α* = 0.05.
(DOCX)

S5 Table **Total encounters at edge effect grids.** Total encounters (including fall 2010, spring 2010 and fall 2011) of amphibians and reptiles at edge transects (n = 6) spanning 40 m into a recent (2–3 yr) clearcut and 60 m into adjacent mature forest.
(DOCX)

S6 Table **Mean encounters of salamanders at harvest effect grids.** Mean encounters (± standard error) per sampling occasion of the most commonly encountered salamander species by treatment type and treatment period.
(DOCX)

S1 Data File **Salamander count data for harvest effect grids.** Salamander counts by grid and sampling occasion at harvest effect grids for the most commonly encountered species (data are rarefied.).
(CSV)

S2 Data File **Downed woody debris data for harvest effect grids.** Volume of DWD as measured each spring at each harvest effect gird.
(CSV)

S3 Data File **Salamander count data for harvest effect grids formatted for program PRESENCE.** Salamander counts for the three most commonly encountered species as formatted for N-mixture models in program PRESENCE.
(CSV)

S4 Data File **Survey covariate data at harvest effect grids formatted for program PRESENCE.** Precipitation, temperature, season, and sample period data at harvest effect grids as formatted for N-mixture models.
(CSV)

S5 Data File **Salamander count data for edge effect grids.** Salamander counts by grid and sampling occasion at edge effect grids for the three most commonly encountered species.
(CSV)

S6 Data File **Downed woody debris data for edge effect grids.** Volume of DWD as measured each spring at each edge effect grid.
(CSV)

S7 Data File **Salamander count data for edge effect grids formatted for program PRESENCE.** Salamander counts for the most commonly encountered species as formatted for N-mixture models in program PRESENCE.
(CSV)

S8 Data File **Survey covariate data at edge effect grids formatted for program PRESENCE.** Precipitation, cover board temperature, season, and soil moisture data at edge effect grids as formatted for N-mixture models.
(CSV)

S9 Data File **Descriptions of variables in supporting information data files.**
(DOCX)
