# Supplementary figures and images for: Effects of Timber Harvests and Silvicultural Edges on Terrestrial Salamanders
Source: PLoS One. 2014 Dec 17;9(12):e114683. doi: 10.1371/journal.pone.0114683 (PMC4269416; doi:10.1371/journal.pone.0114683)

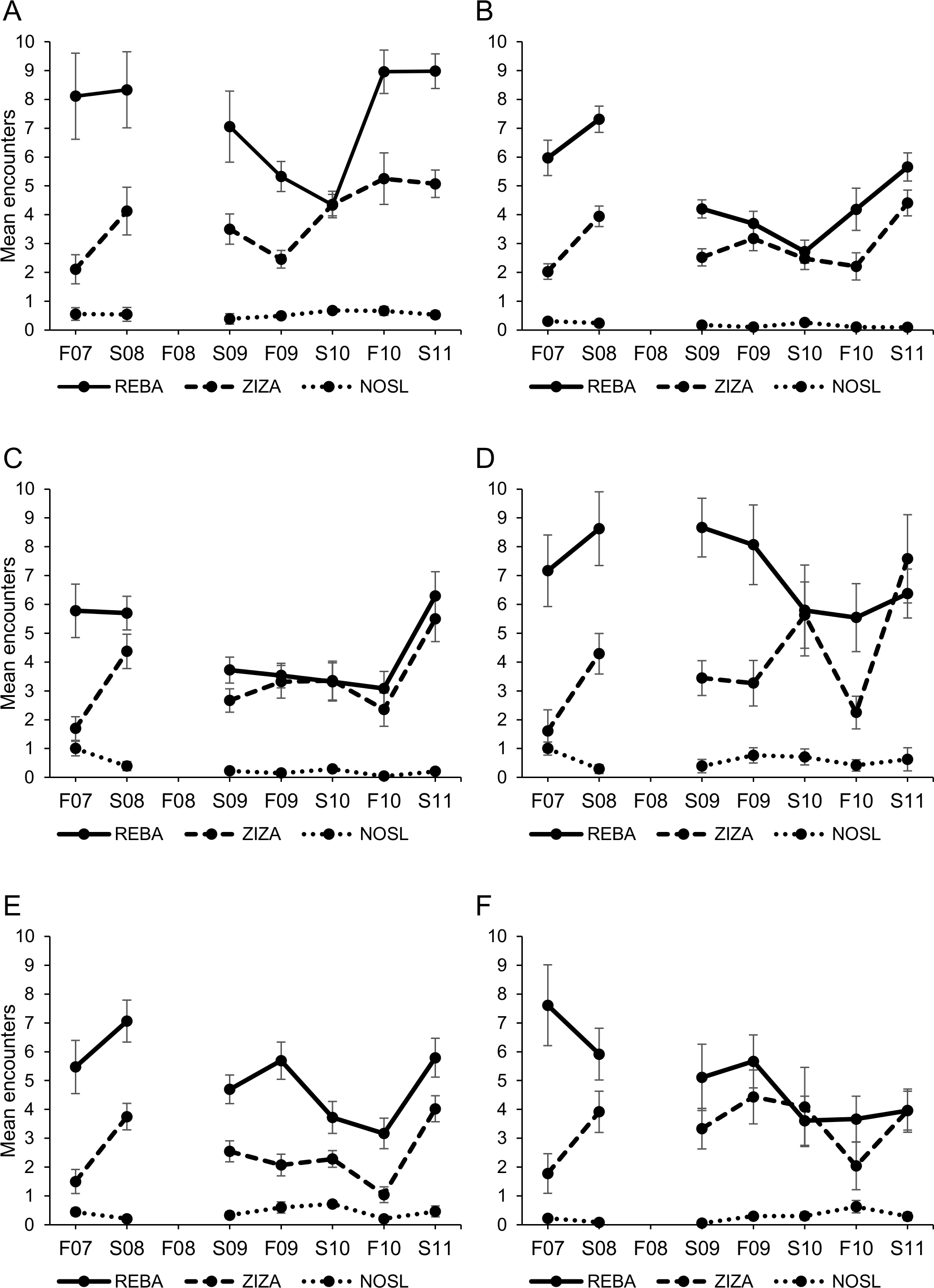

Supplement: S1 Figure — Mean encounters by treatment type and sample period. Mean encounters of red-backed (Plethodon cinereus, REBA), zigzag (P. dorsalis, ZIZA), and northern slimy (P. glutinosus, NOSL) salamanders per sampling occasion by sample period at (A) control, (B) group selection, (C), clearcut, (D) clearcut adjacent, (E) shelterwood and (F) shelterwood adjacent treatment sites. Means are calculated from rarefied data. Error bars represent ± standard error. (TIF) [file pone.0114683.s001.tif]
