# Supplementary material for: Effects of Timber Harvests and Silvicultural Edges on Terrestrial Salamanders
Source: PLoS One. 2014 Dec 17;9(12):e114683. doi: 10.1371/journal.pone.0114683 (PMC4269416; doi:10.1371/journal.pone.0114683)
Supplement: S1 Table — Published studies on salamanders and timber harvests. Studies investigating the effects of silvicultural treatments on terrestrial salamander abundance in North America. (DOCX) [file pone.0114683.s002.docx]

**Table S1.** **Published studies on salamanders and timber harvests.** Studies investigating the effects of silvicultural treatments on terrestrial salamander abundance in North America.^a^

| **Source** | **Location** | **Treatment^b^** | **Method^c^** | **Pre-trt data?** | **Length** | **Effect^d^** |
| --- | --- | --- | --- | --- | --- | --- |
| [22] | NC | CC | ACS | N | 1 yr | – |
| [23] | MO | Un-specified | ACS | N | 2 yrs | – |
| [24] | WA | CC, TH | PF | Y^f^ | 3 yrs | – ^e^ |
| [25] | MO | CC, G, S | PF, MR | Y | 8 yrs | None |
| [29] | VA | CC, G, H, LT, S | V, ACO | Y | 4 yrs | –(CC,G,LT,S) |
| [31] | NY | CC, PC | ACS | N | 4 yrs | –(CC); + (PC^e^) |
| [32] | ME | CC | PF, MR | N | 1 yr | – |
| [33] | NH | Logging | ACO | N | 4 yrs | – |
| [38] | NH | Logging, fire | ACO | N | 1 yr | – |
| [50] | NC | CC | ACS | N | 1 yr | – |
| [51] | NC | CC | V, MR | Y | 15 yrs | – |
| [52] | WV | CC, UN | PF | N | 2 yrs | –(UN) |
| [54] | VA, WV | CC, G, H, LT, S | V | Y | 3-5 yrs | –(CC,G,LT,S) |
| [55] | Ontario | S | ACO | Y | 6 yrs | – |
| [58] | Quebec | CC | ACS, PF, MR | N | 1 yr | – |
| [60] | GA | CC | PF | N | 1 yr | + / – ^e^ |
| This study | IN | CC, G, S | ACO | Y | 5 yrs | –(CC,G) |

^a^Table design modified from deMaynadier and Hunter (1995), Table A1 [21]; this list is not exhaustive.

^b^CC = clearcut; G = group selection; H = herbicide; LT = leave-tree; PC = partial cut; S = shelterwood; TH = thinning; UN = uneven-aged.

^c^ACO = artificial cover object; ACS = area constrained survey; MR = mark-recapture; PF = pitfall traps; V = visual (nighttime, ACS).

^d^Effect of treatment on salamanders; “–” = negative effect; “+” = positive effect; “+ / –” = both positive and negative effects found; effect applies only to treatments listed in parentheses.

^e^Effect varied by species or life stage.

^f^Pre-treatment sampling conducted on thinning harvests but not on clearcut harvests.
