# Supplementary material for: Effects of Timber Harvests and Silvicultural Edges on Terrestrial Salamanders
Source: PLoS One. 2014 Dec 17;9(12):e114683. doi: 10.1371/journal.pone.0114683 (PMC4269416; doi:10.1371/journal.pone.0114683)
Supplement: S2 Table — Total encounters at harvest effect grids. Total encounters of amphibians and reptiles by treatment type and treatment period. (DOCX) [file pone.0114683.s003.docx]

**Table S2.** **Total encounters at harvest effect grids.** Total encounters^a^ of amphibians and reptiles by treatment type^b^ and treatment period.^c^

|  | **Control** | | **Group** | | **CC** | | | **CC adj** | | | **Sh** | | **Sh adj** | | **Total** | |
| --- | --- | --- | --- | --- | --- | --- | --- | --- | --- | --- | --- | --- | --- | --- | --- | --- |
| **n^d^** | **42** | **426** | **165** | **468** | **82** | | **240** | **42** | | **120** | **84** | **227** | **42** | **119** | **457** | **1600** |
| **Species** | **Pre** | **Post** | **Pre** | **Post** | **Pre** | **Post** | | **Pre** | **Post** | | **Pre** | **Post** | **Pre** | **Post** | **Pre** | **Post** |
| *Plethodon cinereus* | 346 | 2904 | 1114 | 1910 | 470 | 955 | | 336 | 823 | | 536 | 1058 | 279 | 528 | 3081 | 8178 |
| *P. dorsalis* | 137 | 1767 | 519 | 1402 | 262 | 833 | | 132 | 531 | | 234 | 539 | 126 | 431 | 1410 | 5503 |
| *P. glutinosus* | 23 | 246 | 44 | 67 | 54 | 43 | | 25 | 72 | | 26 | 107 | 6 | 39 | 178 | 574 |
| *Eurycea cirrigera* | 1 | 1 | 28 | 16 | 3 | 2 | | 1 | 6 | | 11 | 4 | 9 | 8 | 53 | 37 |
| *Notophthalmus viridescens* | 0 | 3 | 0 | 6 | 0 | 2 | | 0 | 1 | | 0 | 1 | 0 | 1 | 0 | 14 |
| *Ambystoma maculatum* | 0 | 0 | 0 | 1 | 2 | 0 | | 0 | 0 | | 0 | 3 | 0 | 1 | 2 | 5 |
| *A. opacum* | 0 | 5 | 0 | 0 | 0 | 0 | | 0 | 0 | | 0 | 0 | 0 | 0 | 0 | 5 |
| *E. longicauda* | 0 | 1 | 0 | 0 | 0 | 3 | | 0 | 0 | | 0 | 0 | 0 | 0 | 0 | 4 |
| *A. jeffersonianum* | 0 | 0 | 0 | 1 | 0 | 0 | | 0 | 0 | | 0 | 0 | 0 | 0 | 0 | 1 |
| unknown salamander spp. | 0 | 1 | 0 | 0 | 0 | 1 | | 0 | 1 | | 0 | 0 | 0 | 0 | 0 | 3 |
| Total salamanders (all species) | 507 | 4928 | 1705 | 3403 | 791 | 1839 | | 494 | 1434 | | 807 | 1712 | 420 | 1008 | 4724 | 14324 |
| *Anaxyrus americanus* | 0 | 1 | 0 | 1 | 0 | 0 | | 0 | 0 | | 0 | 0 | 0 | 1 | 0 | 3 |
| *A. fowleri* | 0 | 0 | 0 | 0 | 0 | 1 | | 0 | 0 | | 0 | 0 | 0 | 0 | 0 | 1 |
| *Plestiodon* spp. | 0 | 6 | 0 | 17 | 0 | 6 | | 0 | 0 | | 1 | 3 | 0 | 5 | 1 | 37 |
| *Diadophis punctatus edwardsii* | 0 | 5 | 2 | 22 | 2 | 10 | | 2 | 0 | | 3 | 5 | 0 | 3 | 9 | 45 |
| *Carphophis amoenus helenae* | 0 | 2 | 1 | 23 | 0 | 10 | | 0 | 2 | | 0 | 2 | 0 | 2 | 1 | 41 |
| *Thamnophis sirtalis sirtalis* | 0 | 0 | 0 | 2 | 0 | 1 | | 0 | 0 | | 0 | 0 | 0 | 0 | 0 | 3 |
| *Storeria dekayi wrightorum* | 0 | 1 | 0 | 0 | 0 | 1 | | 0 | 0 | | 0 | 0 | 0 | 0 | 0 | 2 |
| *Agkistrodon contortrix mokasen* | 0 | 0 | 0 | 1 | 0 | 0 | | 0 | 0 | | 0 | 0 | 0 | 0 | 0 | 1 |
| *Virginia valeriae elegans* | 0 | 0 | 0 | 1 | 0 | 0 | | 0 | 0 | | 0 | 0 | 0 | 0 | 0 | 1 |
| *S. occipitomaculata occipitomaculata* | 0 | 0 | 0 | 0 | 0 | 0 | | 0 | 0 | | 1 | 0 | 0 | 0 | 1 | 0 |
| unknown snake spp. | 0 | 0 | 0 | 0 | 0 | 3 | | 0 | 0 | | 0 | 1 | 0 | 0 | 0 | 4 |

^a^Data were rarefied to reflect equal sampling effort among grids within each sampling season.

^b^CC=clearcut; CC adj=clearcut adjacent; Sh=shelterwood; Sh adj=shelterwood adjacent.

^c^Pre-harvest includes fall 2007 and spring 2008; post-harvest includes spring and fall 2009, spring and fall 2010, and spring 2011.

^d^n = number of sampling occasions (one sampling occasion = a single check of a single grid).
