# Supplementary material for: Effects of Timber Harvests and Silvicultural Edges on Terrestrial Salamanders
Source: PLoS One. 2014 Dec 17;9(12):e114683. doi: 10.1371/journal.pone.0114683 (PMC4269416; doi:10.1371/journal.pone.0114683)
Supplement: S3 Table — Type III fixed effects for analysis of variance Model 1 (data from control sites and group cuts). Asterisks indicate significant effects at α = 0.05. (DOCX) [file pone.0114683.s004.docx]

**Table S3. Type III fixed effects for analysis of variance Model 1 (data from control sites and group cuts).** Asterisks indicate significant effects at *α* = 0.05.

|  | ***P. cinereus*** | | ***P. dorsalis*** | | ***P. glutinosus*** | |
| --- | --- | --- | --- | --- | --- | --- |
| **Effect^a^** | **F** | ***p*** | **F** | ***p*** | **F** | ***p*** |
| T^b^ | 9.06 | 0.027* | 1.37 | 0.282 | 2.61 | 0.173 |
| TP^c^ | 37.97 | <0.001* | 2.58 | 0.112 | 17.83 | <0.001* |
| SP^d^ | 23.50 | <0.001* | 18.76 | <0.001* | 30.07 | <0.001* |
| A^e^ | 2.55 | 0.116 | 17.81 | <0.001* | 8.20 | 0.006* |
| T x TP | 0.92 | 0.342 | 0.00 | 0.990 | 0.18 | 0.670 |
| T x SP | 3.05 | 0.012* | 3.89 | 0.002* | 0.97 | 0.436 |
| T x A | 2.17 | 0.147 | 4.99 | 0.030* | 2.50 | 0.120 |
| A x SP | 3.47 | 0.003* | 2.64 | 0.018* | 1.99 | 0.070 |
| T x A x SP | 1.19 | 0.312 | 1.99 | 0.070 | 1.69 | 0.126 |
| DWD^f^ | 0.42 | 0.520 | 4.69 | 0.032* | 4.42 | 0.037* |

*Significant effect at *α* = 0.05.

^a^Interaction terms are indicated by an ‘x’ between two or more factors.

^b^T = treatment type.

^c^TP = treatment period (pre- or post-harvest).

^d^SP = sample period (fall or spring in a given year).

^e^A = slope aspect (northeast or southwest).

^f^Volume of downed woody debris.
