# Supplementary material for: Effects of Timber Harvests and Silvicultural Edges on Terrestrial Salamanders
Source: PLoS One. 2014 Dec 17;9(12):e114683. doi: 10.1371/journal.pone.0114683 (PMC4269416; doi:10.1371/journal.pone.0114683)
Supplement: S4 Table — Type III fixed effects for analysis of variance Model 2 (data from clearcut, clearcut adjacent, shelterwood, and shelterwood adjacent treatment types). Asterisks indicate significant effects at α = 0.05. (DOCX) [file pone.0114683.s005.docx]

**Table S4. Type III fixed effects for analysis of variance Model 2 (data from clearcut, clearcut adjacent, shelterwood, and shelterwood adjacent treatment types).** Asterisks indicate significant effects at *α* = 0.05.

|  | ***P. cinereus*** | | ***P. dorsalis*** | | ***P. glutinosus*** | |
| --- | --- | --- | --- | --- | --- | --- |
| **Effect^a^** | **F** | ***p*** | **F** | ***p*** | **F** | ***p*** |
| T^b^ | 3.27 | 0.036* | 3.22 | 0.038* | 4.18 | 0.009* |
| TP^c^ | 26.82 | <0.001* | 11.37 | 0.001* | 2.48 | 0.119 |
| SP^d^ | 5.09 | <0.001* | 25.74 | <0.001* | 22.90 | <0.001* |
| A^e^ | 1.23 | 0.278 | 23.03 | <0.001* | 19.82 | <0.001* |
| T x TP | 1.02 | 0.390 | 4.33 | 0.008* | 6.01 | 0.001* |
| T x SP | 1.30 | 0.210 | 1.18 | 0.292 | 1.64 | 0.069 |
| T x A | 0.03 | 0.994 | 5.98 | 0.003* | 2.36 | 0.081 |
| A x SP | 4.53 | <0.001* | 2.15 | 0.052 | 0.53 | 0.783 |
| T x A x SP | 0.94 | 0.534 | 1.16 | 0.305 | 0.50 | 0.957 |
| DWD^f^ | 0.11 | 0.741 | 0.01 | 0.931 | 1.48 | 0.227 |

*Significant effect at *α* = 0.05.

^a^Interaction terms are indicated by an ‘x’ between two or more factors.

^b^T = treatment type.

^c^TP = treatment period (pre- or post-harvest).

^d^SP = sample period (fall or spring in a given year).

^e^A = slope aspect (northeast or southwest).

^f^Volume of downed woody debris.
