# Supplementary material for: Effects of Timber Harvests and Silvicultural Edges on Terrestrial Salamanders
Source: PLoS One. 2014 Dec 17;9(12):e114683. doi: 10.1371/journal.pone.0114683 (PMC4269416; doi:10.1371/journal.pone.0114683)
Supplement: S5 Table — Total encounters at edge effect grids. Total encounters (including fall 2010, spring 2010 and fall 2011) of amphibians and reptiles at edge transects (n = 6) spanning 40 m into a recent (2–3 yr) clearcut and 60 m into adjacent mature forest. (DOCX) [file pone.0114683.s006.docx]

**Table S5.** **Total encounters at edge effect grids.** Total encounters (including fall 2010, spring 2010 and fall 2011) of amphibians and reptiles at edge transects (n = 6) spanning 40 m into a recent (2-3 yr) clearcut and 60 m into adjacent mature forest.

|  |  | **Distance (m)** | | | | | |  |
| --- | --- | --- | --- | --- | --- | --- | --- | --- |
| **Taxon** | **Species** | **-40** | **-20** | **0 (edge)** | **20** | **40** | **60** | **Total** |
| Caudata | *Plethodon dorsalis* | 265 | 219 | 241 | 227 | 218 | 272 | 1442 |
|  | *P. cinereus* | 155 | 174 | 167 | 219 | 189 | 233 | 1137 |
|  | *P. glutinosus* | 20 | 16 | 13 | 20 | 23 | 23 | 115 |
|  | *Ambystoma maculatum* | 2 | 3 | 0 | 0 | 0 | 0 | 5 |
|  | *Eurycea longicauda* | 0 | 0 | 3 | 0 | 1 | 0 | 4 |
|  | *Notophthalmus viridescens* | 2 | 1 | 0 | 0 | 0 | 0 | 3 |
|  | *E. cirrigera* | 0 | 0 | 0 | 0 | 1 | 1 | 2 |
|  | unknown salamander spp. | 1 | 6 | 3 | 3 | 3 | 3 | 19 |
|  | Total salamanders (all species) | 445 | 419 | 427 | 469 | 435 | 532 | 2727 |
| Anura | *Anaxyrus fowleri* | 0 | 2 | 0 | 2 | 0 | 0 | 4 |
|  | *A. americanus* | 1 | 1 | 0 | 0 | 0 | 0 | 2 |
| Squamata | *Diadophis punctatus edwardsii* | 2 | 1 | 1 | 2 | 4 | 0 | 10 |
|  | *Carphophis amoenus helenae* | 1 | 1 | 4 | 1 | 1 | 1 | 9 |
|  | *Plestiodon* spp. | 0 | 0 | 3 | 0 | 2 | 1 | 6 |
