# Supplementary material for: Effects of Timber Harvests and Silvicultural Edges on Terrestrial Salamanders
Source: PLoS One. 2014 Dec 17;9(12):e114683. doi: 10.1371/journal.pone.0114683 (PMC4269416; doi:10.1371/journal.pone.0114683)
Supplement: S6 Table — Mean encounters of salamanders at harvest effect grids. Mean encounters (± standard error) per sampling occasion of the most commonly encountered salamander species by treatment type and treatment period. (DOCX) [file pone.0114683.s007.docx]

**Table S6.** **Mean encounters of salamanders at harvest effect grids.** Mean encounters^a^ (± standard error) per sampling occasion of the most commonly encountered salamander species by treatment type^b^ and treatment period.^c^

|  | **Control** | | **Group** | | **CC** | | **CC adj** | | **Sh** | | **Sh adj** | |
| --- | --- | --- | --- | --- | --- | --- | --- | --- | --- | --- | --- | --- |
| **n^d^** | **42** | **426** | **165** | **468** | **82** | **240** | **42** | **120** | **84** | **227** | **42** | **119** |
| **Species** | **Pre** | **Post** | **Pre** | **Post** | **Pre** | **Post** | **Pre** | **Post** | **Pre** | **Post** | **Pre** | **Post** |
| *Plethodon cinereus* | 8.2 ± 1.0 | 6.8 ± 0.3 | 6.8 ± 0.4 | 4.1 ± 0.2 | 5.7 ± 0.5 | 4.0 ± 0.3 | 8.0 ± 0.9 | 6.9 ± 0.6 | 6.4 ± 0.6 | 4.7 ± 0.3 | 6.6 ± 0.8 | 4.4 ± 0.4 |
| *P. dorsalis* | 3.3 ± 0.5 | 4.1 ± 0.3 | 3.1 ± 0.2 | 3.0 ± 0.2 | 3.2 ± 0.4 | 3.5 ± 0.3 | 3.1 ± 0.5 | 4.4 ± 0.5 | 2.8 ± 0.3 | 2.4 ± 0.2 | 3.0 ± 0.5 | 3.6 ± 0.4 |
| *P. glutinosus* | 0.5 ± 0.2 | 0.6 ± 0.1 | 0.3 ± 0.1 | 0.1 ± 0.0 | 0.7 ± 0.1 | 0.2 ± 0.0 | 0.6 ± 0.1 | 0.6 ± 0.1 | 0.3 ± 0.1 | 0.5 ± 0.1 | 0.1 ± 0.1 | 0.3 ± 0.1 |

^a^Means calculated from rarefied totals that reflect equal sampling effort among grids within each sampling season.

^b^CC=clearcut; CC adj=clearcut adjacent; Sh=shelterwood; Sh adj=shelterwood adjacent.

^c^Pre-harvest includes fall 2007 and spring 2008; post-harvest includes spring and fall 2009, spring and fall 2010, and spring 2011.

^d^n = number of sampling occasions (one sampling occasion = a single check of a single grid).
