# Supplementary material for: Effects of Timber Harvests and Silvicultural Edges on Terrestrial Salamanders
Source: PLoS One. 2014 Dec 17;9(12):e114683. doi: 10.1371/journal.pone.0114683 (PMC4269416; doi:10.1371/journal.pone.0114683)
Supplement: S9 Data File — Descriptions of variables in supporting information data files. (DOCX) [file pone.0114683.s016.docx]

Descriptions of variables in Harvest Effect data files (Data files S1-S2). For more detailed descriptions of sampling methods, see Methods section of text.

| Variable | Description |
| --- | --- |
| Unit | study site within the Hardwood Ecosystem Experiment (HEE); 1-9 |
| Grid | coverboard grid designation; first digit is for round of HEE harvests, second digit is for unit, third and fourth digits are two-digit harvest area designation, letter and number are for grid specific to that harvest area (1101A-1904A3) |
| Treatment (Trt) | Harvest technique; Control; Group; CC=clearcut; CC_adj=clearcut adjacent; Shwd=shelterwood; Shwd_adj=shelterwood adjacent |
| SlopeAzimuth | compass bearing taken at middle of top row of boards at each grid, facing downslope |
| SlopeAspect | categorization of azimuths for ANOVA models; northeast (NE)=316-135; southwest (SW)=136-315 |
| TrtPeriod | Treatment Period; Pre-harvest (Pre) = 2007-2008, Post-harvest (Post) = 2009-2011 |
| Year | Year; 2007, 2008, 2009, 2010, 2011 |
| Season | Season; Fall=Sept-Dec; Spring=Mar-May |
| SamplePeriod | 1=Fall 2007, 2=Spring 2008, 4=Spring 2009, 5=Fall 2009, 6=Spring 2010, 7=Fall 2010, 8=Spring 2011 (Sample Period 3 was Fall 2008, when harvests took place and many grids could not be sampled; this data was not included in analysis) |
| Check | Sampling occasion within a season (numbered consecutively for each grid; not based on date) |
| Date | Date of sampling; format YYMMDD |
| Precip | amount of precipitation (cm) during the 48hrs prior to each check; data from NOAA coop stations in Martinsville and Nashville, IN |
| Temp | Avg daily air temperature (Celsius) during the day of each check; data from NOAA coop stations in Martinsville and Nashville, IN |
| DWD | volume (cu.cm/sq.m) of downed woody debris (diameter>10cm); average of measurements from two transects at each grid; measured once each spring; Spring 2008 values used for Fall 2007, Spring 2008, and Fall 2008; Spring 2009 values used for Spring 2009 and Fall 2009; Spring 2010 values used for Spring 2010, Fall 2010, and for 18 new grids added in Fall 2009 |
| DecayClass | In 2010 and 2011, DWD was categorized by decay class 1-5, with 1 being least decayed and 5 being most decayed (Maser et al. 1979 [43]) |
| DecayV | Volume (cu.cm/sq.m) of downed woody debris (diameter>10cm) in each given decay class (not applicable for years 2007-2009, indicated by '.') |

Descriptions of variables in files for N-mixture models of Harvest Effect data (Data files S3-S4).

| Variable | Description | Type of covariate |
| --- | --- | --- |
| Unit | study site within the Hardwood Ecosystem Experiment (HEE) |  |
| SlopeAzimuth | compass bearing taken at middle of top row of boards at each grid, facing downslope |  |
| SlopeAspect | 1=azimuth 0-90 (northeast); 2=azimuth 95-170 (southeast); 3=azimuth 180-270 (southwest); 4=azimuth 282-350 (northwest) | site covariate |
| Treatment | 1=Control 2=Group 3=Clearcut 4=Clearcut adjacent 5=Shelterwood 6=Shelterwood adjacent | site covariate |
| DWDpre | volume (cu.cm/sq.m) of downed woody debris (diameter>10cm) as measured in fall 2007 (pre-harvest) | site covariate |
| DWDpost | volume (cu.cm/sq.m) of downed woody debris (diameter>10cm); average of measurements from spring 2009, spring 2010, and spring 2011 (post-harvest) | site covariate |
| SamplePeriod | 1=Fall2007, 2=Spring2008, 3=Spring 2009, 4=Fall2009, 5=Spring 2010, 6=Fall2010, 7=Spring2011 |  |
| Year | Year |  |
| Season | Season; Fall=Sept-Dec; Spring=Mar-May | survey covariate |
| Survey period | two week period within a sample period during which a grid was checked; a few records (<10) had two consecutive checks conducted within the same survey period, but an exception was made in these instances and the survey numbering was altered (the record was not deleted but assigned a survey period slightly outside the date range) |  |
|  | Fall ‘07: 1=9/10-23; 2=9/24-10/7; 3=10/8-21; 4=10/22-11/4; 5=11/5-18; 6=11/19-12/4 |  |
|  | Spring ‘08: 1=3/3-16; 2=3/17-30; 3=3/31-4/13; 4=4/14-27 |  |
|  | Fall ‘08: 1=9/22-10/5; 2=10/6-19; 3=10/20-11/2; 4=11/3-16; 5=11/17-30 |  |
|  | Spring ‘09: 1=3/2-15; 2=3/16-29; 3=3/30-4/9; 4=4/10-26; 5=4/27-5/11 |  |
|  | Fall ‘09: 1=9/7-20; 2=9/21-10/4; 3=10/5-18; 4=10/19-11/1; 5=11/2-15 |  |
|  | Spring ‘10: 1=3/13-28; 2=3/29-4/11; 3=4/12-25; 4=4/26-5/9 |  |
|  | Fall ‘10; 1=9/6-25; 2=9/26-10/7; 3=10/8-19; 4=10/20-11/1; 5=11/2-14; 11/15-30 |  |
|  | Spring ‘11: 1=2/28-3/13; 2=3/14-27; 3=3/28-4/10; 4=4/11-24; 5=4/25-5/8 |  |
| Site | coverboard grid designation; first digit is for round of HEE harvests, second digit is for unit, third and fourth digits are for harvest area, letter and number are for grid specific to that harvest area; designations with an "H" were added partway through the study to replace a grid which was reclassified after harvests as described in the Methods section |  |
| Precip | amount of precipitation (cm) during the 48hrs prior to each check; data from NOAA coop stations in Martinsville and Nashville, IN | survey covariate |
| Temperature | Avg daily air temperature (Celsius) during the day of each check; data from NOAA coop stations in Martinsville and Nashville, IN | survey covariate |
| - | Grid was not surveyed during given survey period |  |

Descriptions of variables in Edge Effect data files (Data files S5-S6).

| Variable | Description |
| --- | --- |
| Unit | study site within the Hardwood Ecosystem Experiment (HEE); 3, 6, 9 |
| Transect | designation for grouping of six grids across edge gradient; 1302T, 1304T, 1601T, 1603T, 1901T, 1904T |
| Distance | distance (m) to edge of harvest; negative values indicate distance into clearcut, positive values indicate distance into forest; -40, -20, 0 (edge), 20, 40, 60 |
| SlopeAspect | general slope face of transect; NE=northeast; SW=southwest |
| Year | Year; 2010, 2011 |
| Season | Season; Fall=Sept-Dec; Spring=Mar-May |
| SamplePeriod | year and season combination; 1=spring 2010; 2=fall 2010; 3=spring 2011 |
| Check | sampling occasion, numbered in chronological order |
| Date | Date of sampling for each check of each grid; format YYMMDD |
| ToD | Time of day during which a grid (distance) was checked; 1=0700-1000; 2=1001-1300; 3=1301-1600; 4=1601-1900. |
| Precip | amount of precipitation (cm) during the 48hrs prior to each check; data from NOAA coop stations in Martinsville and Nashville, IN |
| AirTemp | Mean ambient temperature (Celsius) during the period of the day (ToD) during which a grid was checked, as recorded by digital ibutton dataloggers deployed near the ground at each grid |
| ACOTemp | Mean temperature (Celsius) under ACOs during the period of the day (ToD) during which a grid was checked, as recorded by digital ibutton dataloggers glued under an ACO at each grid |
| Soil | Average soil moisture as measured at five points within each grid on each sampling occasion (using a soil probe to 10 cm) |
| Canopy | Average percent canopy cover as measured at five points within each grid on one occasion each sampling period (using a spherical densiometer) |
| Leaf | Average leaf litter depth as measured at five points within each grid on one occasion each sampling period |
| DWD | volume (cu.cm/sq.m) of downed woody debris (diameter>10cm); measured once each spring; Spring 2010 values used also for Fall 2010, |
| DecayClass | In 2010 and 2011, DWD was categorized by decay class 1-5, with 1 being least decayed and 5 being most decayed (Maser et al. 1979 [43]) |
| DecayV | Volume (cu.cm/sq.m) of downed woody debris (diameter>10cm) in each given decay class (not applicable for years 2007-2009, indicated by '.') |

Descriptions of variables in files for N-mixture models of Edge Effect data (Data files S7-S8).

| Variable | Description | Type of covariate |
| --- | --- | --- |
| Unit | study site within the Hardwood Ecosystem Experiment (HEE); 3, 6, 9 |  |
| Transect | designation for grouping of six grids across edge gradient; 1302T, 1304T, 1601T, 1603T, 1901T, 1904T |  |
| Distance | distance (m) to edge of harvest; negative values indicate distance into clearcut, positive values indicate distance into forest |  |
| Distance coded | 0=60m; 1=40m; 2=20m; 3=0m; 4=-20m; 5=-40m (coded for program PRESENCE) | site covariate |
| SlopeAspect | general slope face of transect; 1=northeast; 2=southwest | site covariate |
| DWD | volume (cu.cm/sq.m) of downed woody debris (diameter>10cm); average of measurements taken in spring 2010 and spring 2011 | site covariate |
| AvgCanopy | Average percent canopy cover as measured at five points within each grid on one occasion each sampling period (using a spherical densiometer); used the average of measurements taken in spring 2010, fall 2010, and spring 2011 | site covariate |
| AvgLeaf | Average leaf litter depth as measured at five points within each grid on one occasion each sampling period; used average of measurements taken spring 2010, fall 2010, and spring 2011 | site covariate |
| SamplePeriod | year and season combination; 1=spring 2010; 1=fall 2010; 3=spring 2011 |  |
| Year | Year |  |
| Season | Season; 1=Fall (Sept-Dec); 2=Spring (Mar-May) | survey covariate |
| Survey | survey #; 1-15 |  |
| Site | coverboard grid designation; combination of transect ID and distance to edge |  |
| Precip | amount of precipitation (cm) during the 48hrs prior to each check; data from NOAA coop stations in Martinsville and Nashville, IN | survey covariate |
| ACOTemp | Mean temperature (Celsius) under ACOs during the period of the day during which a grid was checked, as recorded by digital ibutton dataloggers glued under an ACO at each grid | survey covariate |
| Soil | Average soil moisture as measured at five points within each grid on each sampling occasion (using a soil probe to 10 cm) | survey covariate |

Salamander species codes and scientific names.

| Species code | Scientific Name |
| --- | --- |
| REBA | *Plethodon cinereus* |
| ZIZA | *P. dorsalis* |
| NOSL | *P. glutinosus* |

**Reference**

Maser C, Anderson RG, Cromack K Jr., Williams JT, Martin RE (1979) Dead and down woody material. In: Thomas JW, editor. Wildlife Habitats in Managed Forests: the Blue Mountains of Oregon and Washington, Agricultural Handbook 553. Washington, DC: USDA Forest Service. pp. 78–95.
